# Proline-Based Carbamates as Cholinesterase Inhibitors †

**DOI:** 10.3390/molecules22111969

**Published:** 2017-11-14

**Authors:** Hana Pizova, Marketa Havelkova, Sarka Stepankova, Andrzej Bak, Tereza Kauerova, Violetta Kozik, Michal Oravec, Ales Imramovsky, Peter Kollar, Pavel Bobal, Josef Jampilek

**Affiliations:** 1 Department of Chemical Drugs, Faculty of Pharmacy, University of Veterinary and Pharmaceutical Sciences, Palackeho 1, 612 42 Brno, Czech Republic; F14042@vfu.cz (M.H.); pavelbobal@yahoo.com (P.B.); 2 Department of Biological and Biochemical Sciences, Faculty of Chemical Technology, University of Pardubice, Studentska 573, 532 10 Pardubice, Czech Republic; sarka.stepankova@upce.cz; 3 Institute of Chemistry, University of Silesia, Szkolna 9, 40-007 Katowice, Poland; andrzej.bak@us.edu.pl; 4 Department of Human Pharmacology and Toxicology, Faculty of Pharmacy, University of Veterinary and Pharmaceutical Sciences, Palackeho 1, 612 42 Brno, Czech Republic; tereza.kauerova@gmail.com (T.K.); kollarp@vfu.cz (P.K.); 5 Department of Synthesis Chemistry, Faculty of Mathematics, Physics and Chemistry, University of Silesia, Szkolna 9, 40-007 Katowice, Poland; violetta.kozik@us.edu.pl; 6 Global Change Research Institute CAS, Belidla 986/4a, 603 00 Brno, Czech Republic; oravec.m@czechglobe.cz; 7 Institute of Organic Chemistry and Technology, Faculty of Chemical Technology, University of Pardubice, Studentska 573, 532 10 Pardubice, Czech Republic; ales.imramovsky@upce.cz; 8 Department of Pharmaceutical Chemistry, Faculty of Pharmacy, Comenius University, Odbojarov 10, 832 32 Bratislava, Slovakia

**Keywords:** proline, carbamates, in vitro cholinesterase inhibition, in vitro cytotoxicity assay, CoMSA, IVE-PLS, molecular docking study

## Abstract

Series of twenty-five benzyl (2*S*)-2-(arylcarbamoyl)pyrrolidine-1-carboxylates was prepared and completely characterized. All the compounds were tested for their in vitro ability to inhibit acetylcholinesterase (AChE) and butyrylcholinesterase (BChE), and the selectivity of compounds to individual cholinesterases was determined. Screening of the cytotoxicity of all the compounds was performed using a human monocytic leukaemia THP-1 cell line, and the compounds demonstrated insignificant toxicity. All the compounds showed rather moderate inhibitory effect against AChE; benzyl (2*S*)-2-[(2-chlorophenyl)carbamoyl]pyrrolidine-1-carboxylate (IC_50_ = 46.35 μM) was the most potent agent. On the other hand, benzyl (2*S*)-2-[(4-bromophenyl)-] and benzyl (2*S*)-2-[(2-bromophenyl)carbamoyl]pyrrolidine-1-carboxylates expressed anti-BChE activity (IC_50_ = 28.21 and 27.38 μM, respectively) comparable with that of rivastigmine. The *ortho*-brominated compound as well as benzyl (2*S*)-2-[(2-hydroxyphenyl)carbamoyl]pyrrolidine-1-carboxylate demonstrated greater selectivity to BChE. The in silico characterization of the structure–inhibitory potency for the set of proline-based carbamates considering electronic, steric and lipophilic properties was provided using comparative molecular surface analysis (CoMSA) and principal component analysis (PCA). Moreover, the systematic space inspection with splitting data into the training/test subset was performed to monitor the statistical estimators performance in the effort to map the probability-guided pharmacophore pattern. The comprehensive screening of the AChE/BChE profile revealed potentially relevant structural and physicochemical features that might be essential for mapping of the carbamates inhibition efficiency indicating qualitative variations exerted on the reaction site by the substituent in the 3′-/4′-position of the phenyl ring. In addition, the investigation was completed by a molecular docking study of recombinant human AChE.

## 1. Introduction

An amide (–CONH–) and/or carbamate (–OCONH–) group is present in a number of clinically used drugs [1] and pesticides [2]. Both terminations can be variously substituted, which results in a privileged structural fragment [3,4]. These moieties interact with a number of enzymes/receptors and, by means of these target sites, they are able to affect the biological response. Therefore, the reason for the widespread occurrence of amides and carbamates among new biologically active compounds is obvious [5,6,7,8,9,10,11,12,13,14,15]. Carbamate-like compounds can be considered as essential cholinesterase inhibitors (ChEIs) [1,16,17,18]. Acetyl-(AChE, EC 3.1.1.7) and butyryl-cholinesterases (BChE, EC 3.1.1.8) are two important cholinesterases (ChEs) that occur in the human body. ChEs belong to the group of serine hydrolases [19]. The major role of AChE is to catalyse the hydrolysis of acetylcholine (ACh) in cholinergic synapses, while BChE can hydrolyse ACh as well as other esters; nevertheless, it seems its concentration in brain is especially important at Alzheimer’s disease (AD) [20,21]. The inhibition of both enzymes causes an increase in the ACh concentration in cholinergic synapses and can subsequently affect a number of pathogenic processes. ChEIs are used in the treatment of various neuromuscular disorders and have provided the first generation of drugs for the treatment of Alzheimer’s disease, myasthenia gravis and glaucoma. An increase in the concentration of ACh can result in an alleviation of the symptoms of these diseases [22].

AD is an irreversible, progressive brain disorder that slowly destroys memory and thinking skills, and eventually the ability to carry out the simplest tasks [23]; moreover, the AD is the most common cause of dementia among older adults. It can be stated that it is the most devastating central nervous system disorder, especially in Western civilization. Worldwide, nearly 44 million people have AD or related dementia, and more than 100 million people worldwide are estimated to be affected by AD by 2050 [24,25]. Although the etiology of the AD is not yet entirely known, several conditions are believed to have important roles in the pathogenesis of this disease; they include aggregation and accumulation of amyloid-β deposits, oxidative stress and low levels of ACh [26]. In the AD brain, AChE levels decrease, while BChE levels are reportedly increased or unchanged, with changes becoming more pronounced during the disease course. Strategies that increase the ACh level show symptomatic efficacy in AD treatment [21]; therefore competitive ChEIs, such as galantamine and rivastigmine, as well as non-competitive ChEIs tacrine and donepezil are clinically used for relieving AD. In addition, memantine, a *N*-methyl-d-aspartate (NMDA) receptor antagonist, was also approved by the FDA for relieving AD [25]. Thus, research of new ChEIs may be valuable for further progress in the treatment of AD. Recently, new preferential or BChE selective inhibitors [26,27,28] as well as various multifunctional anti-Alzheimer agents [29,30,31,32] were reported.

This is a follow-up paper to the paper devoted to recently reported carbamates as potential ChEIs [16,17,18,33] and dealing with synthesis and ChE inhibiting properties of benzyl (2*S*)-2-(arylcarbamoyl)pyrrolidine-1-carboxylates **1**–**9c**. In the context of our previously described *N*-heterocycles, simple modifications of proline, an atypical α-amino acid, were investigated. The rational production of properties supported by computers is basically regarded as a preliminary stage or an “intuitive roadmap” on the path from a hit to a drug candidate. A pool of computer-assisted drug design (CADD) procedures, including multidimensional quantitative-structure activity relationships (mD-QSAR), diffused quickly into computational medicinal chemistry getting more and more popular in the rational drug discovery [34].

The in silico characterization of the structure–inhibitory potency for the set of proline-based carbamates considering electronic, steric and lipophilic properties was provided using comparative molecular surface analysis (CoMSA) and principal component analysis (PCA) [35]. Moreover, the systematic space inspection with splitting data into the training/test subset was performed to monitor the statistical estimators performance in the effort to map the probability-guided pharmacophore pattern. Consequently, a pseudo-consensus 3D-QSAR approach was applied to retrieve an “average” pharmacophore hypothesis by investigating the best models for training/test subpopulations to specify the potentially important factors contributing to the inhibitory activity of potential ChEIs. In addition, the investigation of these positional isomers of proline-based carbamates was completed by a molecular docking study of recombinant human AChE (rhAChE). Thus, the comprehensive screening of the AChE/BChE profile revealed the potentially relevant structural and physicochemical features that might be essential for mapping of the carbamates inhibition efficiency, indicating qualitative variations exerted on the reaction site by a substituent in the 3′-/4′-position of the phenyl ring.

## 2. Results

### 2.1. Chemistry

**Scheme 1:**
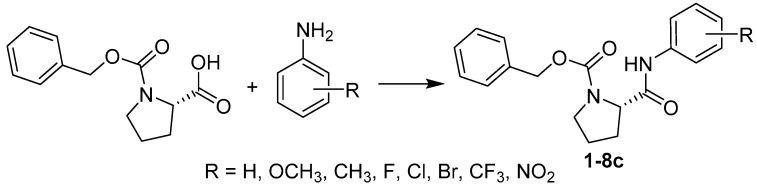
Synthesis of benzyl (2*S*)-2-(arylcarbamoyl)pyrrolidine-1-carboxylate **1**–**8c**. Reagents and conditions: propylphosphonic anhydride, dry ethyl acetate, 80 °C, argon atmosphere.

From conventional and emerging chemical approaches for amide bond formation [3,36,37,38,39,40] and from the methods which have been previously used in our laboratories [8,41,42,43], we have selected the procedure where propylphosphonic anhydride (T3P) has been used as a coupling agent [43]. The application of this one-pot reaction to commercially available (2*S*)-1-[(benzyloxy)carbonyl]-pyrrolidine-2-carboxylic acid and a wide variety of substituted anilines led to the formation of corresponding substituted anilides **1**–**8c** (see Scheme 1) that were isolated in high yields and high purity.

**Scheme 2:**
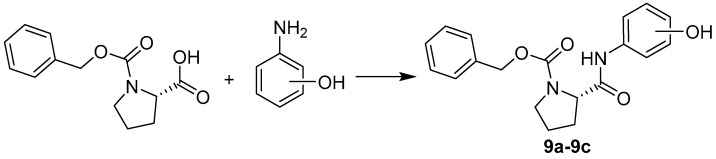
Synthesis of benzyl (2*S*)-2-[(hydroxyphenyl)carbamoyl]pyrrolidine-1-carboxylates **9a**–**9c**. Reagents and conditions: triethylamine, tetrahydrofuran, ethyl chloroformate, room temperature, argon atmosphere.

Unfortunately, the method with T3P failed in the case of aminophenols. To overcome this problem with formation of many side products, the modified Mukayama’s method [44,45] with use of ethyl chloroformate as an activation reagent in the presence of base has been successfully applied (see Scheme 2). Hydroxy-substituted anilides **9a**–**9c** were isolated in high yields and sufficient purity.

### 2.2. In Vitro Evaluation of *AChE*- and *BChE*-Inhibiting Activity with *CoMSA* and *SMV* Procedure

All prepared carbamates were tested for their inhibition of AChE and BChE. The activities of the compounds were compared with the internal standards rivastigmine and galantamine. These standards were chosen because of their different structures. While rivastigmine is a classical acylating pseudo-reversible carbamate ChEI that inhibits both AChE and BChE, galanthamine is a non-acylating competitive reversible ChEI as well as an allosteric ligand at nicotinic ACh receptors. The choice of these reference drugs with different mechanisms of action can provide relevant results. The results are summarized in Table 1 and expressed as 50% inhibitory concentration (IC_50_ (μM)), or the concentration of inhibitor required for 50% inhibition of the mentioned enzymes.

The principal aim of the ligand-based investigation was the comparative study of the molecular surface using the CoMSA approach to model in vitro activity observed for a set of proline-based carbamates as potential AChE/BCHE inhibitors. The findings of inhibiting potency modeling (AChE and BChE) were correlated with surface descriptors regarding multiple training/test subsets and (in)dependent variables used, respectively. Unfortunately, the $q_{cv}^{2}$ performance of AChE and BChE profile for the entire proline-based carbamates dataset **1**–**9c** in the training dataset is not satisfactory for CoMSA models ($q_{cv}^{2}$ < 0.5), irrespective of the map size (20 × 20–50 × 50) or applied template molecules **1** and **7a**–**c**. Obviously, a model predictive power cannot be evaluated just by goodness of data fitting with the cross-validated leave-one-out (CV–LOO) procedure [46]. Thus, the external validation with splitting the molecule subset into a set of training/test collections was performed to evaluate the model predictive ability with SDEP and $q^{2}{}_{test}$ statistics as well. The CoMSA performance for models divided arbitrarily into training/test subsets in 2:1 ratio (18/7) ranked according to the molecule inhibitory activity (AChE and BChE), regarding *ortho-*, *meta-*, and *para*-substitution (or their combinations), indicated pretty poor model abilities accompanied with poor model predictive power. Moreover, the Kennard–Stone procedure on dependent variables was applied to split representatively the collection of data into training/test subsets, however the statistical characteristics of CoMSA models for AChE/BChE was not noticeably improved.

The obtained findings confirmed that the separation of objects into training/test subgroups is not a trivial issue. Hence, an additional assessment, namely Stochastic Model Validation (SMV), has been performed as a kind of “perturbation” procedure to investigate the data structure [47]. Thus, the fluctuations of the statistical estimators during CoMSA AChE and BChE modeling were scrutinized, as the original dataset of 25 molecules was recurrently sampled into 18/7 training/test series (fraction 2/3 to 1/3). In this case, it was technically viable to investigate the entire pool of systematically generated training/test populations ($C_{25}^{8}$ ≈ 10^6^). The observed profile for $q_{cv}^{2}$ vs. $q^{2}{}_{test}$ distribution confirms the intuitive interpretation of $q_{cv}^{2}$/$q^{2}{}_{test}$ fluctuation pattern, where the areas of higher modeling ability within the training set can be depicted for AChE as well as BChE potency ($q_{cv}^{2}$ ≥ 0.75). On the other hand, the preferential choice of objects into the training set that easily fit into the model is accompanied with the decline in the predictive performance for the remaining ones, which confirms the dichotomic nature of $q_{cv}^{2}$/$q^{2}{}_{test}$ parameters, where high value of $q_{cv}^{2}$ does not automatically imply a high model predictability [48]. It should be emphasized that the great advantage of the QSAR/QSPR paradigm lies not in the extrapolation as stated Hansch [49].

**Figure 1:**
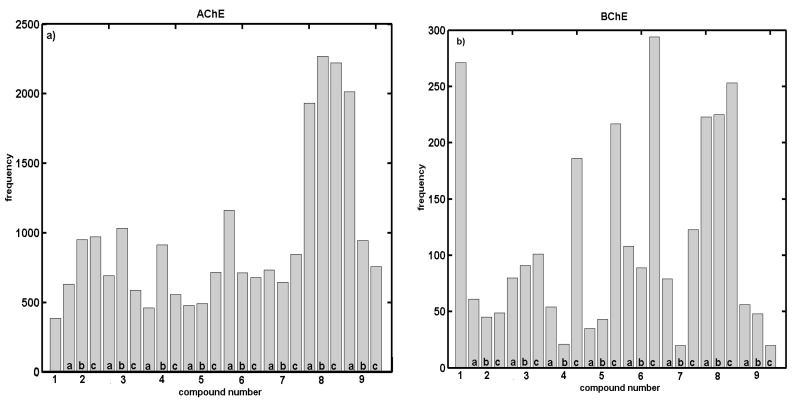
Histograms specify number of individual compounds appearing in test set within regions of $q_{cv}^{2}$ > 0.5 for training set against $q_{test}^{2}$ L–7–O CV for modeling: AChE (**a**); and BChE (**b**) potency of proline-based carbamates derivatives with CoMSA method.

Additionally, Figure 1a,b depicts the molecule selection frequency into the test subset in the function of the compound number, while sampling the best models ($q_{cv}^{2}$ ≥ 0.50). Surprisingly, a relatively smooth compound distribution within the training/test subpopulations is disrupted by outnumbering of molecules **8a**–**c** for AChE vs. **1**, **4**–**6c**, **8a**–**c** for BChE profile (common compounds **8a**–**c**). Generally speaking, the indicated molecules are mainly *para-*substituted isomers. Interestingly, the BChE inhibitory activities for the positional isomers increase progressively and can be ranked according to the rough relation, where *ortho* > *meta* > *para*, which partly explains the preferential selection of *para-*positioned molecules into the test subset.

**Figure 2:**
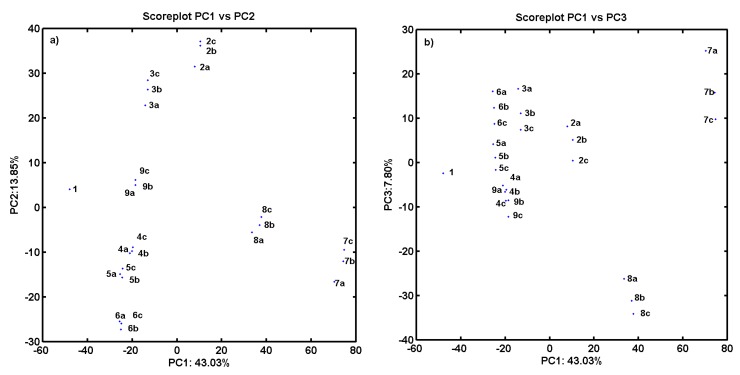
Projection of carbamates **1**–**9c** on plane defined by: first vs. second (**a**); and first vs. third (**b**) principal components for Dragon descriptors.

As an additional experiment, the PCA procedure for an ensemble of descriptors retrieved from Dragon 6.0 software (Nuance Communications, Burlington, MA, USA) has been applied to the analyzed compounds. The final dataset was arranged in matrix *X*_25×2821_ with rows representing molecules (called objects) and columns presenting numerical parameters (called variables) for further analysis. PCA was applied to visualize major differences in the performance of investigated molecules with respect to their structure and inhibitory profile. The analysis was performed for centered and standardized data. The PCA model with first four PCs described 71.88% of the total data variance, while the first three PCs account for 64.68%. The respective score plots are presented in Figure 2a,b. An analysis of score plots PC1 vs. PC2 in Figure 2a and PC1 vs. PC3 in Figure 2b indicates that, basically, proline-based carbamate derivatives can be classified into groups considering structural data—the positional isomers are generally grouped together. PC1, which describes 43.03% of total variance, reveals the major variations between compounds **1** and **7a**–**c** (used as template molecules in CoMSA analysis) and all the remaining ones. Indeed, molecule **1** (object no. 1) is unmodified (only hydrogen substituent) compared to its derivatives containing CF_3_ group (compounds **7a**–**c**) characterized by the highest value of the volume descriptors provided by the Sybyl–X 2.0 software. Not surprisingly, the visualization of objects encoded by a set of Dragon descriptors (parameters) on the plane defined by PC1 vs. PC2 indicates a similar relationship within the ensemble of carbamate analogs along the first principal component, especially for molecules **4**–**6c** excluded from the best models. Interestingly, this group of compounds is characterized by large negative values on PC2 as illustrated in Figure 2a.

**Figure 3:**
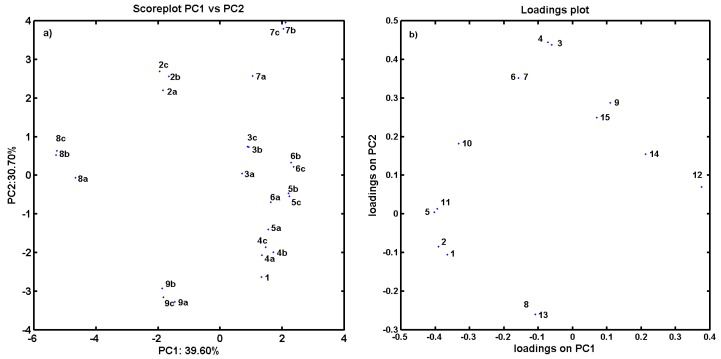
Projection of carbamates **1**–**9c** on plane specified by first vs. second principal component (**a**); and corresponding loadings score for Sybyl descriptors (**b**).

**Figure 4:**
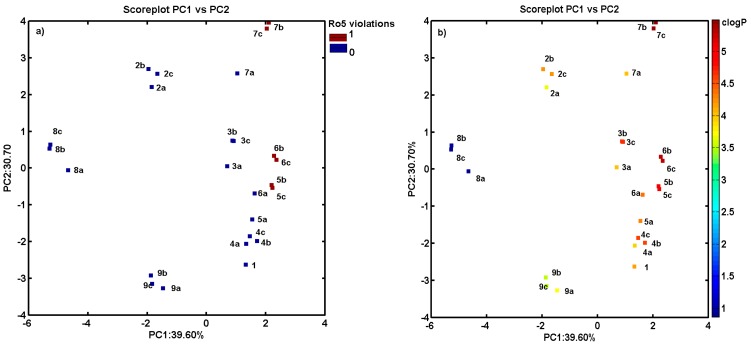
Projection of carbamates **1**–**9c** on plane defined by first vs. second principal component with: number of Ro5 rule violations (**a**); and molecule lipophilicity profile (**b**). Colors code the number of Ro5 violations and value of calculated lipophilicity.

Similarly, 15 parameters (variables) produced by the Sybyl software were collected including count, volume, surface, Ro5 and lipophilicity descriptors (see Table 1 and Table S1 in Supplementary materials) to examine the variations within the ensemble of carbamate derivatives. The compression of the data slowly increased with the number of PCs that were included. The first two PCs account for 70.30% of the total data variance, and it increases to ca. 81.47% for next four PCs. The score and the corresponding loading plots are presented in Figure 3a,b. The projection of objects on the plane defined by PC1 vs. PC2 component confirmed the observed previously tendency to cluster compounds **4**–**6c** together with noticeable dissimilarities to positional isomers **8** (containing NO_2_ substituent). On the basis of the loading plots (Figure 3b), it could be concluded that the uniqueness of the above molecules was caused by the positively correlated variables describing the molecular prosperities (e.g., clogP, MW or polar volume along PC1). Moreover, compounds **5**–**7** were also clustered more or less according to Lipinski rule of five (Ro5) violations (isomers *meta*/*para*) as displayed in Figure 4a.

Basically, one can expect that the lipophilic profile for molecules can be related to their chemical structure, therefore structurally similar compounds, namely chemotypes, should have similar property features. The detailed inspection of compound lipophilicity color-coded accordingly to calculated values of clogP for objects projected on the plane specified by two first principal components (PC1 vs. PC2) confirmed this tendency as observed in Figure 4b for compound separation detected along PC1 (compounds **5**–**7** with clogP > 5).

### 2.3. Consensus-Based *3D* Pharmacophore Mapping

Thousands of highly correlated topologic/topographic-based descriptors are normally generated in mD-QSAR studies; however, the informative variable selection is not an indispensable pre-processing procedure to prune the input assemble of generated descriptors.

**Figure 5:**
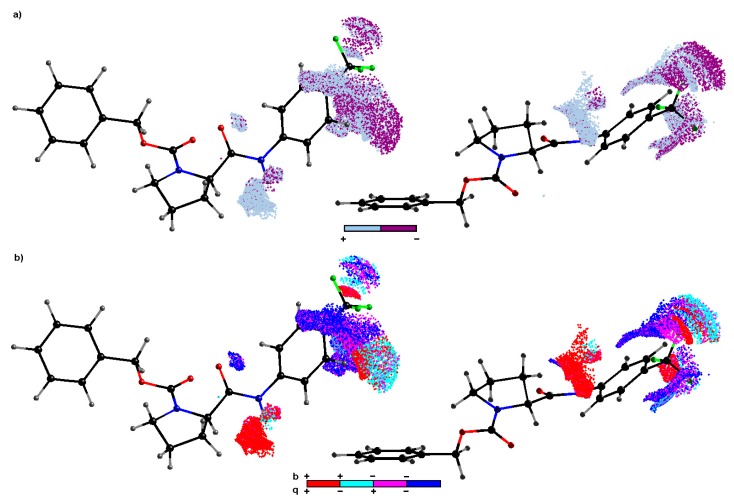
CoMSA IVE–PLS monitored for chosen 18/7 training/test set samplings. Molecular plots show the spatial sectors of the greatest contribution into the BChE inhibitory potency. Colors code the sign of influence (**a**). Four possible combinations of mean charge and correlation coefficient are color–coded for charge descriptors (**b**). Compound **7c** was plotted in two different orientations as a reference molecule.

The subsequent level of variable elimination was applied in order to generate meaningful and predictive models. The recursive IVE-PLS procedure was employed as a “sieve” to identify structural descriptors having the highest individual weightings to the biological activity [50]. Hence, all 18/7 training/test samplings specified for regions with pretty high model abilities ($q_{cv}^{2}$ ≥ 0.5) were selected to produce an “average” pharmacophore. Regarding the number of objects, the maximum number of PLS components considered for the model generation was truncated to 7. Thus, the columns annotated with the highest stability for each of the randomly chosen models were identified using the IVE-PLS methodology. Basically, a minor improvement of the $q_{cv}^{2}$ performance was observed, while columns from the data matrix assigned with the lowest value of abs(mean(b)/std(b)) were extracted; however, the model predictability monitored by $q^{2}{}_{test}$ remains stable for a considerable range of variables eliminated in the majority of training/test samplings. The moment of $q_{cv}^{2}$ deterioration determines the number of the relevant columns, hence the backward column elimination is recurrently repeated until the optimal number of variables included within the model is accomplished. The cumulative sum of common columns for all investigated AChE or BChE models was calculated and normalized to the range of (0–1). Initially, the group of columns with the value above the pre-chosen cut-off of 0.4 was selected, but the spatial pattern illustrated in Figure 5 was generated by further filtering of 50% of CoMSA descriptors with relatively small statistical significance for the inhibitory activities. The relative contribution of each variable is weighted by the magnitude and the sign of the corresponding regression coefficient; therefore colors code the sign of the descriptor impact on compound potency. A visual inspection of the key pharmacophore patterns can provide direct knowledge about the interaction mode that increases/decreases the compound activity. The sign of influence is color-coded depicting not only regions with positive and/or negative activity contribution, but also four possible combinations of the mean charge/correlation coefficient.

The dark spheres in Figure 5a indicate the patterns potentially detrimental for the BChE inhibitory potency (mainly due to steric hindrance or electrostatic factors), while the bright polyhedral specify the 3D areas where atom/substituent is predicted to be positioned in order to enhance the compound’s inhibition profile. In fact, large regions with suggested favorable contribution appear in the close proximity to the nitrogen attached directly to the carbonyl moiety (peptide-bond-like motif in the scaffold). It suggests that the positively charged hydrogen bonded to this nitrogen atom can be significant for the affinity of the inhibitor molecule, while interacting non-covalently with a macromolecular site (hydrogen bond donor) as indicated by the corresponding positive regression coefficient pool in Figure 5b. Moreover, the obtained findings demonstrate the importance of the side chain R directly attached to the phenyl ring (see Table 1); especially positions 3′ and/or 4′ seem to be a crucial structural and physicochemical factor for the activity maintenance of the tested compounds. As a matter of fact, the mixed (negative/positive) steric contribution to the inhibitory efficiency is observed in Figure 5a; however, the increase of bulkiness at position 4′ of the phenyl ring seem to be unfavorable structural variations, which can partially explain the lower BChE potency of *para* isomers. In general, the negatively charged atoms/groups in *para* position primarily favorably contribute (negative regression coefficients) to the inhibition efficiency as illustrated in Figure 5b. On the other hand, the spatially allowed areas attributed by the positive regression coefficient of CoMSA models are occupied by the positively-charged carbon atom of CF_3_ or nitrogen of NO_2_ fragments, which corresponds quite well with the enhanced inhibitory activities (AChE & BChE < 100) observed for CF_3_ and NO_2_ substituted derivatives within the *ortho* population.

The stochastic SMV protocol for the pharmacophore visualization based on the consensus 3D QSAR modeling with satisfactory statistical characteristics provides the spatial map of chemical groups/atoms potentially relevant for increasing/decreasing the activity profile of the proline-based carbamates as potential ChEIs.

### 2.4. Molecular Docking Study

In the target-guided QSAR procedures the complementary (bio)effector binding mode is retrieved based on the intrinsic dependence of atomic coordinates of both receptor and ligand in the binding/active site, while the target spatial arrangement of atoms is available [51]. The adopted spatial distribution of the ligand property space is mediated by the corresponding mapping of target steric, electronic or lipophilic patterns. The promising site-directed QSAR methodology called docking can be employed when the macromolecular geometry or at least good homology models are available. Molecular docking is a method extensively used in the structure based drug design (SBDD); however; this method does not always provide quantitative correlations between in silico calculations and actual activity assays [52]. On the other hand, the SBDD methodology is a sophisticated tool in the lead optimization and virtual screening of the hits retrieved from the ligand database in flash docking. In particular, the structures of human acetylcholinesterase in a complex with pharmacologically relevant drugs (e.g., galanthamine or rivastigmine) were a matter of previous extensive studies [53] that allowed us to make a comparison with ligand-based protocols; however, the detailed investigation of host–target interactions is beyond the scope of this paper.

**Figure 6:**
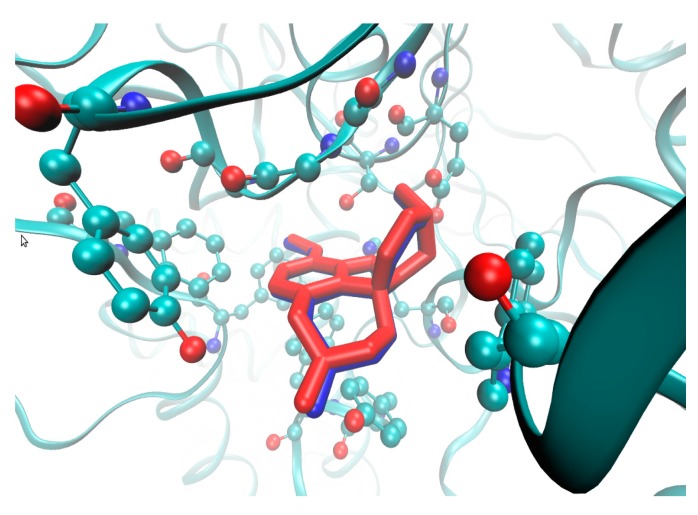
Structure of rhAChE bound with galanthamine (blue) and docked with AutoDock Vina (red) in active site of enzyme chain A (depicted amino acid residues of Trp86, Gly120-122, Tyr124, Glu202, Ser203, Phe295, Phe297, Tyr337, Phe338, His447).

The crystallographic data of rhAChE with the catalytic core specified at higher resolution of 2.4 Å in the liganded state (holo) with galanthamine was obtained from 4EY6 PDB entry. Subsequently, the drug molecule was successfully (re)docked into the active site of the enzyme chain A using AutoDock Vina program (Scripps Research Institute, La Jolla, CA, USA) [54], which represents a flexible platform for the rapid database screening as presented in Figure 6.

**Figure 7:**
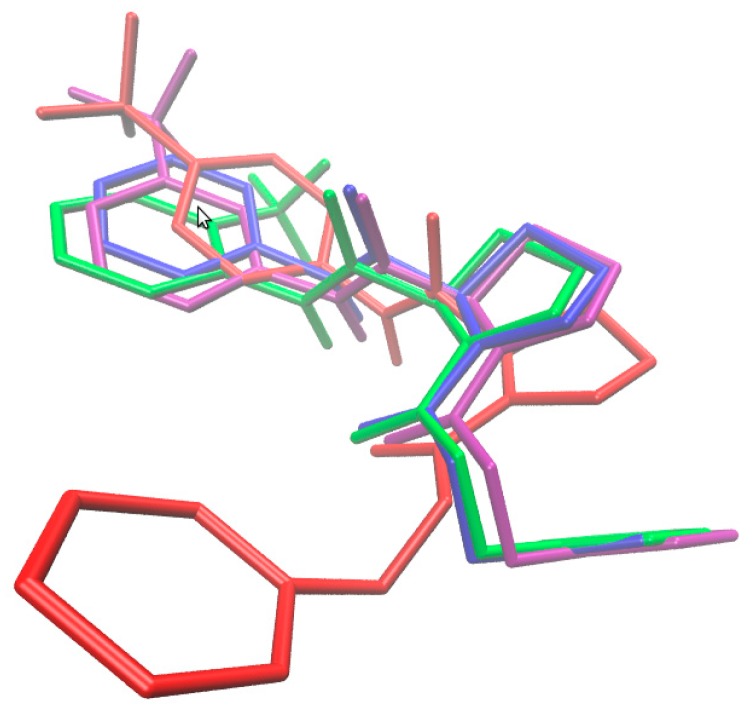
Comparison of poses for molecules **1** (blue), **7a** (green), **7b** (magenta) and **7c** (red) in active site of enzyme chain A.

Moreover, the attempt to investigate the spatial host–rhAChE patterns within the active site of chain A was taken for the population of proline-based carbamates (**1**–**9c**) and compared with galanthamine interacting mode. Firstly, the comparison of particular conformations and mutual orientations (poses) for compounds **1** and **7a**–**c** is illustrated in Figure 7, indicating relevant structural variations in the catalytic binding site. It seems that *para*-substituted derivatives exert potentially different impact on the enzyme reaction site, which is in line with our receptor-independent findings.

**Figure 8:**
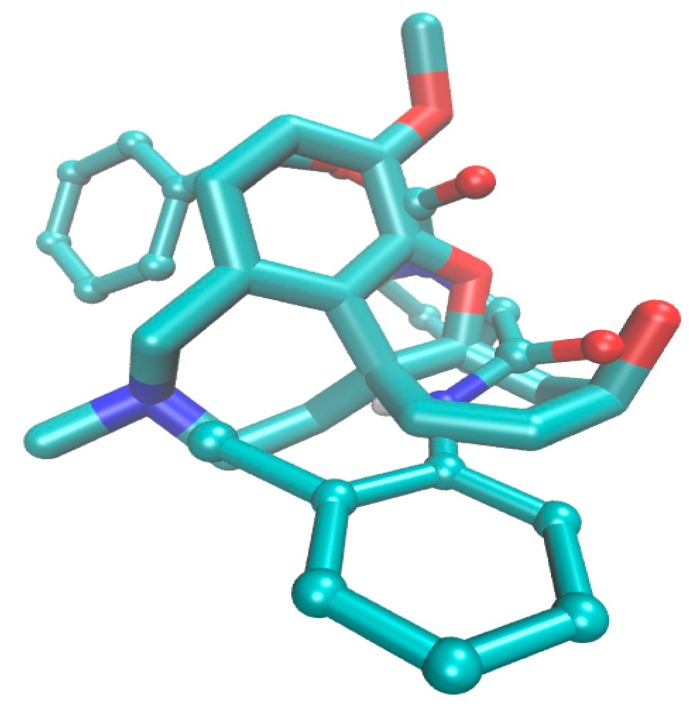
Models of galanthamine (displayed as sticks) and molecule **5a** (depicted as ball-and-sticks) in rhAChE catalytic core with nitrogen atom (blue) and oxygen (red), respectively.

Surprisingly, despite a noticeable structural differences between galanthamine and the most active AChE molecule **5a** in the set of proline-based carbamates, a similar spatial distribution of the negatively charged atoms (nitrogen and oxygen) is observed in the catalytic core as illustrated in Figure 8. In fact, regions in the close proximity to the nitrogen atom linked directly to the carbonyl motif were also indicated to have favorable contribution to the inhibitory profile in the consensus CoMSA study.

Moreover, the obtained results demonstrate the importance of the side chain R, especially for *ortho*-positioned analogs directly attached to the phenyl ring. Interestingly, a chlorine atom can contribute to hydrogen bond interactions with Tyr337 of chain A as postulated in the case of the galanthamine binding mode [53]. Roughly speaking, the inhibition activity profile for the positional isomers can be partially explained by the possibility of hydrogen bond interactions in the catalytic core ranked according to *ortho* > *meta* > *para* substitution. It seems that an increase of bulkiness at the *para* position of the phenyl ring is unfavorable for the inhibitory potency of the investigated carbamates as observed also in the pharmacophore study.

Ironically, most ligand based results do not seem to be consistent with the structure based findings; therefore we should beware of docking [55]. On the other hand, a systematic screening of multifaceted drug-receptor bonding/repulsive forces using target-tailored procedures conjugated with consensus pharmacophore mapping can lead towards an intelligent drug delivery platform.

### 2.5. In Vitro Cytotoxicity Assay

The preliminary in vitro screening of the cytotoxicity of the most effective compounds was performed using the human monocytic leukemia THP-1 cell line, as described previously [8]. The cytotoxicity was evaluated as the IC_50_ value (compound concentration causing 50% inhibition of cell population proliferation) (see Table 1). A compound is considered as cytotoxic when it demonstrates a toxic effect on cells at the concentration up to 10 μM [56], and the highest tested concentration that was used for the toxicity assay was three times this value. Treatment with 30 μM of the discussed compounds did not lead to a significant lethal effect on THP-1 cells. Based on these observations, it can be concluded that the discussed compounds can be considered as non-toxic agents for subsequent design of novel drugs.

## 3. Materials and Methods

### 3.1. General Methods

All reagents were purchased from Aldrich. TLC experiments were performed on alumina-backed silica gel 60 F254 plates (Merck, Darmstadt, Germany). The plates were illuminated under UV (254 nm). The melting points were determined on Böetius PHMK 05 (Franz Küstner Nachf, Dresden, Germany) and are uncorrected. The purity of final compounds was analyzed by a Dionex Ultimate 3000 (Thermo Scientific) HPLC system controlled through the Chromeleon^®^ Chromatography Data System (version 7.2, Thermo Scientific, Waltham, MA USA). The separation was performed on a YMC-Tiart C_18_ (3 μm, 150 mm × 2 mm) column (Agilent Technologies, Waldbronn, Germany). Mobile phase consists of water and acetonitrile in the ratio of 40:60. The total flow rate was 0.2 mL/min; the injection volume was 1 μL; and the column temperature was maintained at 35 °C. The detection wavelength of 210 nm was chosen. The purity of individual compounds was calculated as the average of relative peak areas in the chromatograms of the sample solution. The measurement of optical rotations was carried on Automatic polarimeter AA-10 (Optical Activity, Ramsey, UK). The concentration of samples is given in g/100 mL. Infrared (IR) spectra were recorded on a Smart MIRacle^TM^ ATR ZnSe for Nicolet^TM^ Impact 410 FT-IR Spectrometer (Thermo Scientific). The spectra were obtained by accumulation of 256 scans with 2 cm^−1^ resolution in the region of 4000–600 cm^−1^. All ^1^H, ^19^F and ^13^C spectra were recorded on a JEOL ECZR 400 MHz NMR spectrometer (400 MHz for ^1^H, 101 MHz for ^13^C and 376 MHz for ^19^F, Jeol, Tokyo, Japan) in DMSO-*d*_6_. Chemical shifts (δ) are reported in ppm. In ^1^H-NMR spectra there are very broad signals due to interconversion of carbamate rotamers. In ^13^C and ^19^F NMR spectra two signals for two rotamers are observed in most cases [57]. High-resolution mass spectra were measured using a high-performance liquid chromatograph Dionex UltiMate^®^ 3000 (Thermo Scientific) coupled with a LTQ Orbitrap XL^TM^Hybrid Ion Trap-Orbitrap Fourier Transform Mass Spectrometer (Thermo Scientific) with injection into HESI II in the positive or negative mode.

### 3.2. Synthesis

General Procedure for Synthesis of Carbamates **1**–**8c**: To a mixture of an appropriate aniline (3.01 mmol, 1.5 equivalent) and *N*-benzyloxycarbonyl-l-proline (2.06 mmol) in dry ethyl acetate (5 mL) propylphosphonic anhydride (T3P, 50% solution in toluene, 4.01 mmol of T3P, 2 equivalent) was added dropwise. The reaction mixture was stirred overnight at 80 °C under atmosphere of argon. Reaction was monitored by thin layer chromatography. After disappearance of the starting material, water (10 mL) and ethyl acetate (10 mL) were added. The pH of the reaction mixture was adjusted to pH 6 using 5 M NaOH solution. Organic layer was separated, washed twice with water and dried with anhydrous Na_2_SO_4_, and solvent was evaporated under reduced pressure. The residue was triturated with diethyl ether/petroleum ether or purified by column chromatography on silica gel (hexane/ethyl acetate; *v*/*v*, 2:1) to give **1**–**8c**. All the studied compounds are presented in Table 1.

*Benzyl (2S)-2-(phenylcarbamoyl)pyrrolidine-1-carboxylate* (**1**) [45]. Yield 80%; m.p. 140–141 °C; HPLC pur. 99.39%; ${\left[\alpha \right]}_{D}^{25}$: −123.3 (*c* 1.0, CHCl_3_) ^1^H-NMR (DMSO-*d*_6_) δ: 10.03 (d, *J* = 4.4 Hz, 1H), 7.59 (dd, *J* = 7.2, 5.6 Hz, 2H), 6.97–7.45 (m, 8H), 4.88–5.17 (m, 2H), 4.25–4.47 (m, 1H), 3.21–3.63 (m, 2H), 2.09–2.38 (m, 1H), 1.75–2.06 (m, 3H); ^13^C-NMR (DMSO-*d*_6_) δ: 170.99, 170.70, 153.69, 139.00, 138.92, 136.95, 136.77, 128.64, 128.62, 128.04, 127.47, 126.86, 123.28, 123.20, 119.32, 119.15, 65.91, 65.84, 60.47, 59.95, 47.22, 46.57, 31.28, 30.20, 23.96, 23.16; IR (cm^−1^): 3270, 2874, 1698, 1666, 1549, 1425, 1355, 1245, 1179, 1124, 986, 905, 758, 698; HR-MS: for C_19_H_20_N_2_O_3_ [M + H]^+^ calculated 325.1547 *m*/*z*, found 325.1548 *m*/*z*.

*Benzyl (2S)-2-[(2-methoxyphenyl)carbamoyl]pyrrolidine-1-carboxylate* (**2a**) [57]. Yield 67%; m.p. 74–76 °C; HPLC pur. 99.57%; ${\left[\alpha \right]}_{D}^{25}$: −107.0 (*c* 1.0, CHCl_3_); ^1^H-NMR (DMSO-*d*_6_) δ: 9.20 (s, 1H), 7.90–8.05 (m, 1H), 6.99–7.45 (m, 7H), 6.85–6.96 (m, 1H), 4.92–5.17 (m, 2H), 4.57 (t, *J* = 9.3 Hz, 1H), 3.69–3.89 (m, 3H), 3.23–3.58 (m, 2H), 2.04–2.32 (m, 1H), 1.76–2.03 (m, 3H); ^13^C-NMR (DMSO-*d*_6_) δ: 171.04, 170.58, 153.80, 149.51, 136.84, 128.37, 128.05, 127.42, 126.88, 124.38, 121.68, 121.68, 121.08, 120.21, 119.43, 111.09, 65.98, 65.84, 60.40, 59.82, 55.70, 47.22, 46.63, 31.35, 29.85, 23.96, 23.11; IR (cm^−1^): 3274, 2963, 1705, 1684, 1536, 1414, 1351, 1251, 1120, 1025, 955, 745, 698; HR-MS: for C_20_H_22_N_2_O_4_ [M + H]^+^ calculated 355.1652 *m*/*z*, found 355.1654 *m*/*z*.

*Benzyl (2S)-2-[(3-methoxyphenyl)carbamoyl]pyrrolidine-1-carboxylate* (**2b**). Yield 79%; m.p. 118–120 °C; HPLC pur. 98.89%; ${\left[\alpha \right]}_{D}^{25}$: −115.6 (*c* 1.0, CHCl_3_); ^1^H-NMR (DMSO-*d*_6_) δ: 10.03 (d, *J* = 3.7 Hz, 1H), 7.29–7.41 (m, 3H), 7.08–7.26 (m, 5H), 6.60–6.68 (m, 1H), 4.92–5.13 (m, 2H), 4.30–4.40 (m, 1H), 3.70–3.75 (m, 3H), 3.30–3.56 (m, 2H), 2.11–2.33 (m, 1H), 1.78–1.99 (m, 3H); ^13^C-NMR (DMSO-*d*_6_) δ: 171.07, 170.79, 159.48, 154.02, 153.68, 140.21, 140.12, 136.95, 136.79, 129.45, 129.43, 128.38, 128.04, 127.49, 126.86, 111.57, 111.39, 108.89, 108.82, 104.99, 104.80, 65.93, 65.85, 60.50, 60.00, 54.97, 54.95, 47.22, 46.58, 31.27, 30.20, 23.96, 23.15; IR (cm^−1^): 3262, 2944, 1697, 1671, 1597, 1550, 1425, 1349, 1200, 1121, 1049, 955, 879, 768, 700; HR-MS: for C_20_H_22_N_2_O_4_ [M + H]^+^ calculated 355.1652 *m*/*z*, found 355.1653 *m*/*z*.

*Benzyl (2S)-2-[(4-methoxyphenyl)carbamoyl]pyrrolidine-1-carboxylate* (**2c**) [45,58]. Yield 58%; m.p. 122–124 °C; HPLC pur. 98.84%; ${\left[\alpha \right]}_{D}^{25}$: −126.0 (*c* 1.0, CHCl_3_) ^1^H-NMR (DMSO-*d*_6_) δ: 9.88 (d, *J* = 4.8 Hz, 1H), 7.44–7.58 (m, 2H), 7.10–7.42 (m, 5H), 6.82–6.95 (m, 2H), 4.91–5.12 (m, 2H), 4.26–4.38 (m, 1H), 3.72 (d, *J* = 2.4 Hz, 3H), 3.27–3.57 (m, 2H), 2.08–2.32 (m, 1H), 1.71–2.00 (m, 3H); ^13^C-NMR (DMSO-*d*_6_) δ: 170.50, 170.20, 155.25, 155.16, 154.02, 153.71, 136.96, 136.81, 132.17, 132.04, 128.37, 128.08, 127.46, 126.86, 120.91, 120.68, 113.75, 65.89, 65.82, 60.41, 59.90, 55.15, 47.22, 46.58, 31.31, 30.23, 23.97, 23.17; IR (cm^−1^): 3274, 2940, 1696, 1668, 1550, 1509, 1444, 1411, 1358, 1244, 1167, 1119, 1035, 836, 766, 697; HR-MS: for C_20_H_22_N_2_O_4_ [M + H]^+^ calculated 355.1652 *m*/*z*, found 355.1655 *m*/*z*.

*Benzyl (2S)-2-[(2-methylphenyl)carbamoyl]pyrrolidine-1-carboxylate* (**3a**) [58]. Yield 73%; m.p. 115–117 °C; HPLC pur. 98.99%; ${\left[\alpha \right]}_{D}^{25}$: −123.0 (*c* 1.0, CHCl_3_); ^1^H-NMR (DMSO-*d*_6_) δ: 9.39 (br. s., 1H), 7.03–7.46 (m, 10H), 5.00–5.15 (m, 2H), 4.43 (t, *J* = 9.4 Hz, 1H), 3.27–3.61 (m, 2H), 2.04–2.35 (m, 3H + 1H), 1.77–2.03 (m, 3H); ^13^C-NMR (DMSO-*d*_6_) δ: 170.93, 170.60, 154.19, 153.82, 136.98, 136.84, 136.03, 135.90, 132.29, 130.19, 128.36, 128.21, 127.20, 125.83, 125.45, 65.94, 60.31, 59.67, 47.18, 46.58, 31.46, 30.16, 24.01, 23.17, 17.69, 17.59; IR (cm^−1^): 3258, 2948, 1715, 1662, 1528, 1416, 1353, 1130, 995, 737, 697; HR-MS: for C_20_H_22_N_2_O_3_ [M + H]^+^ calculated 339.1703 *m*/*z*, found 339.1706 *m*/*z*.

*Benzyl (2S)-2-[(3-methylphenyl)carbamoyl]pyrrolidine-1-carboxylate* (**3b**) [58]. Yield 65%; m.p. 101–103 °C; HPLC pur. 99.68%; ${\left[\alpha \right]}_{D}^{25}$: −122.5 (*c* 1.0, CHCl_3_); ^1^H-NMR (DMSO-*d*_6_) δ: 9.96 (d, *J* = 6.9 Hz, 1H), 7.29–7.49 (m, 4H), 7.07–7.28 (m, 4H), 6.81–6.93 (m, 1H), 4.90–5.14 (m, 2H), 4.35 (ddd, *J* = 15.7, 8.3, 3.0 Hz, 1H), 3.31–3.56 (m, 2H), 2.13–2.32 (m, 3H + 1H), 1.77–1.98 (m, 3H); ^13^C-NMR (DMSO-*d*_6_) δ: 171.02, 170.73, 154.08, 153.76, 138.97, 138.86, 137.91, 137.85, 137.01, 136.85, 128.46, 128.12, 127.54, 126.94, 124.05, 123.96, 120.00, 119.77, 116.61, 116.40, 65.99, 65.89, 60.52, 60.01, 47.27, 46.63, 31.34, 30.26, 24.01, 23.20, 21.22; IR (cm^−1^): 3274, 2874, 1670, 1557, 1425, 1351, 1203, 1125, 951, 749, 696; HR-MS: for C_20_H_22_N_2_O_3_ [M + H]^+^ calculated 339.1703 *m*/*z*, found 339.1706 *m*/*z*.

*Benzyl (2S)-2-[(4-methylphenyl)carbamoyl]pyrrolidine-1-carboxylate* (**3c**) [58]. Yield 76%; m.p. 143–146 °C; HPLC pur. 99.81%; ${\left[\alpha \right]}_{D}^{25}$: −122.0 (*c* 1.0, CHCl_3_); ^1^H-NMR (DMSO-*d*_6_) δ: 9.94 (d, *J* = 5.5 Hz, 1H), 7.42–7.52 (m, 2H), 7.28–7.41 (m, 2H), 7.07–7.26 (m, 5H), 4.92–5.13 (m, 2H), 4.35 (ddd, *J* = 11.4, 8.2, 3.1 Hz, 1H), 3.35–3.57 (m, 2H), 2.21–2.29 (m, 3H + 1H), 1.79–2.01 (m, 3H); ^13^C-NMR (DMSO-*d*_6_) δ: 170.76, 170.46, 154.01, 153.70, 136.96, 136.80, 136.40, 132.18, 129.01, 128.98, 128.37, 128.06, 127.46, 126.85, 119.40, 119.17, 65.90, 65.83, 60.44, 59.92, 47.22, 46.57, 31.30, 30.22, 23.96, 23.15, 20.43; IR (cm^−1^): 3270, 2874, 1695, 1668, 1514, 1428, 1352, 1251, 1123, 955, 819, 749, 697; HR-MS: for C_20_H_22_N_2_O_3_ [M + H]^+^ calculated 339.1703 *m*/*z*, found 339.1705 *m*/*z*.

*Benzyl (2S)-2-[(2-fluorophenyl)carbamoyl]pyrrolidine-1-carboxylate* (**4a**) [45]. Yield 96%; m.p. 113–115 °C; HPLC pur. 98.66%; ${\left[\alpha \right]}_{D}^{25}$: −110.6 (*c* 1.0, CHCl_3_); ^1^H-NMR (DMSO-*d*_6_) δ: 9.85 (s, 1H), 7.76–7.92 (m, 1H), 7.35–7.40 (m, 2H), 7.29–7.34 (m, 1H), 7.18–7.29 (m, 3H), 7.11–7.18 (m, 2H), 4.96–5.13 (m, 2H), 4.49–4.57 (m, 1H), 3.40–3.56 (m, 2H), 2.15–2.32 (m, 1H), 1.80–1.98 (m, 3H); ^13^C-NMR (DMSO-*d*_6_) δ: 171.50, 171.13, 154.10, 153.76 (d, ^1^*J_CF_* = 244.2 Hz, 1C), 153.68, 153.54 (d, ^1^*J_CF_* = 244.2 Hz, 1C), 136.97, 136.79, 128.37, 128.08, 127.75, 127.48, 127.44, 126.94, 125.97 (d, ^2^*J_CF_* = 22.0 Hz, 1C), 125.89 (d, ^2^*J_CF_* = 21.7 Hz, 1C), 125.39 (d, ^3^*J_CF_* = 7.5 Hz, 1C), 125.38 (d, ^3^*J_CF_* = 7.2 Hz, 1C), 125.23 (d, ^3^*J_CF_* = 7.5 Hz, 1C), 125.22 (d, ^3^*J_CF_* = 7.2 Hz, 1C), 124.29 (d, ^2^*J_CF_* = 21.7 Hz, 1C), 124.21 (d, ^4^*J_CF_* = 3.5 Hz, 2C), 124.15 (d, ^2^*J_CF_* = 22.0 Hz, 1C), 115.48, 115.35, 65.94, 65.87, 60.10, 59.54, 47.18, 46.58, 31.34, 30.14, 23.93, 23.11; ^19^F NMR (DMSO-*d*_6_) δ: −124.91–−124.76 (m, 1F), −124.75–−124.53 (m, 1F); IR (cm^−1^): 3266, 2878, 1672, 1537, 1421, 1353, 1254, 1122, 986, 762, 744, 696; HR-MS: for C_19_H_19_FN_2_O_3_ [M − H]^−^ calculated 341.1307 *m*/*z*, found 341.1309 *m*/*z*.

*Benzyl (2S)-2-[(3-fluorophenyl)carbamoyl]pyrrolidine-1-carboxylate* (**4b**). Yield 96%; m.p. 82–84 °C; HPLC pur. 98.93%; ${\left[\alpha \right]}_{D}^{25}$: −109.3 (*c* 1.0, CHCl_3_); ^1^H-NMR (DMSO-*d*_6_) δ: 10.26 (s, 1H), 7.59 (t, *J* = 11.2 Hz, 1H), 7.26–7.43 (m, 4H), 7.06–7.25 (m, 3H), 6.83–6.94 (m, 1H), 4.89–5.15 (m, 2H), 4.29–4.41 (m, 1H), 3.26–3.58 (m, 2H), 2.12–2.35 (m, 1H), 1.76–2.03 (m, 3H); ^13^C-NMR (DMSO-*d*_6_) δ: 171.40, 171.12, 162.10 (d, ^1^*J_CF_* = 241.2 Hz, 1C), 162.09 (d, ^1^*J_CF_* = 241.4 Hz, 1C), 154.04, 153.65, 140.73 (d, ^3^*J_CF_* = 6.9 Hz, 1C), 140.59 (d, ^3^*J_CF_* = 6.6 Hz, 1C), 136.92, 136.73, 130.33 (d, ^3^*J_CF_* = 9.7 Hz, 1C), 130.28 (d, ^3^*J_CF_* = 9.4 Hz, 1C), 128.38, 128.01, 127.79, 127.48, 127.46, 126.94, 115.05 (d, ^4^*J_CF_* = 2.5 Hz, 1C), 114.80 (d, ^4^*J_CF_* = 2.7 Hz, 1C), 109.76 (d, ^2^*J_CF_* = 21.3 Hz, 1C), 109.69 (d, ^2^*J_CF_* = 21.3 Hz, 1C), 106.12 (d, ^2^*J_CF_* = 26.2 Hz, 1C), 105.95 (d, ^2^*J_CF_* = 26.5 Hz, 1C), 65.97, 65.91, 60.52, 60.01, 47.22, 46.58, 31.21, 30.15, 23.98, 23.17; ^19^F NMR (DMSO-*d*_6_) δ: −112.13–−112.00 (m, 1 F), −111.99–−111.88 (m, 1F); IR (cm^−1^): 3503, 3021, 1671, 1607, 1432, 1356, 1297, 1205, 1122, 961, 874, 769, 699; HR-MS: for C_19_H_19_FN_2_O_3_ [M − H]^−^ calculated 341.1307 *m*/*z*, found 341.1309 *m*/*z*.

*Benzyl (2S)-2-[(4-fluorophenyl)carbamoyl]pyrrolidine-1-carboxylate* (**4c**) [45]. Yield 96%; m.p. 122–124 °C; HPLC pur. 99.53%; ${\left[\alpha \right]}_{D}^{25}$: −116.8 (*c* 1.0, CHCl_3_); ^1^H-NMR (DMSO-*d*_6_) δ: 10.09 (s, 1H), 7.60 (td, *J* = 9.6, 5.0 Hz, 2H), 7.08–7.41 (m, 7H), 4.90–5.14 (m, 2H), 4.27–4.39 (m, 1H), 3.37–3.57 (m, 2H), 2.11–2.33 (m, 1H), 1.78–2.01 (m, 3H); ^13^C-NMR (DMSO-*d*_6_) δ: 170.92, 170.63, 158.01 (d, ^1^*J_CF_* = 239.8 Hz, 1C), 157.95 (d, ^1^*J_CF_* = 240.1 Hz, 1C), 154.03, 153.69, 136.94, 136.75, 135.39 (d, ^4^*J_CF_* = 2.5 Hz, 1C), 135.29 (d, ^4^*J_CF_* = 2.5 Hz, 1C), 128.37, 128.05, 127.77, 127.47, 126.90, 121.09 (d, ^3^*J_CF_* = 7.5 Hz, 2C), 120.91 (d, ^3^*J_CF_* = 7.7 Hz, 2C), 115.22 (d, ^2^*J_CF_* = 22.1 Hz, 2C), 115.19 (d, ^2^*J_CF_* = 22.1 Hz, 2C), 65.94, 65.87, 60.46, 59.94, 47.22, 46.58, 31.25, 30.19, 23.98, 23.17; ^19^F NMR (DMSO-*d*_6_) δ: −119.35–−119.25 (m, 1F), −119.23–−119.13 (m, 1F); IR (cm^−1^): 3278, 3080, 1668, 1555, 1426, 1354, 1214, 1196, 1128, 959, 741, 696; HR-MS: for C_19_H_19_FN_2_O_3_ [M − H]^−^ calculated 341.1307 *m*/*z*, found 341.1309 *m*/*z*.

*Benzyl (2S)-2-[(2-chlorophenyl)carbamoyl]pyrrolidine-1-carboxylate* (**5a**). Yield 85%; m.p. 76–78 °C; HPLC pur. 98.84%; ${\left[\alpha \right]}_{D}^{25}$: −106.0 (*c* 1.0, CHCl_3_); ^1^H-NMR (DMSO-*d*_6_) δ: 9.55–9.64 (m, 1H), 7.71 (d, *J* = 7.6 Hz, 1H), 7.48 (t, *J* = 6.5 Hz, 2H), 7.24–7.41 (m, 5H), 7.20 (t, *J* = 7.3 Hz, 1H), 4.99–5.13 (m, 2H), 4.47–4.55 (m, 1H), 3.48–3.56 (m, 1H), 3.44 (t, *J* = 7.3 Hz, 1H), 2.16–2.33 (m, 1H), 1.96–2.03 (m, 1H), 1.81–1.96 (m, 2H); ^13^C-NMR (DMSO-*d*_6_) δ: 171.27, 170.89, 154.30, 153.79, 136.92, 136.81, 134.66, 134.55, 129.41, 128.19, 127.46, 127.13, 126.56, 125.85, 66.02, 65.94, 60.35, 59.70, 47.18, 46.60, 31.31, 29.97, 23.96, 23.11; IR (cm^−1^): 3266, 1677, 1524, 1423, 1354, 1291, 1181, 1121, 1041, 955, 761, 698; HR-MS: for C_19_H_19_ClN_2_O_3_ [M − H]^−^ calculated 357.1011 *m*/*z*, found 357.1017 *m*/*z*.

*Benzyl (2S)-2-[(3-chlorophenyl)carbamoyl]pyrrolidine-1-carboxylate* (**5b**). Yield 79%; m.p. 101–103 °C; HPLC pur. 99.08%; ${\left[\alpha \right]}_{D}^{25}$: −117.8 (c 1.0, CHCl_3_); ^1^H-NMR (DMSO-*d*_6_) δ: 10.23 (br. s., 1H), 7.76–7.85 (m, 1H), 7.44 (dd, *J* = 18.2, 8.2 Hz, 1H), 7.28–7.39 (m, 3H), 7.16–7.26 (m, 2H), 7.06–7.15 (m, 2H), 5.03–5.14 (m, 1H), 4.93 (d, *J* = 12.9 Hz, 1H), 4.29–4.38 (m, 1H), 3.42–3.56 (m, 2H), 2.17–2.31 (m, 1H), 1.80–1.98 (m, 3H); ^13^C-NMR (DMSO-*d*_6_) δ: 171.43, 171.15, 154.06, 153.65, 140.39, 136.72, 132.99, 130.35, 128.39, 128.02, 127.48, 126.96, 123.04, 122.97, 118.91, 118.70, 117.73, 117.56, 65.98, 65.91, 60.55, 60.04, 47.22, 46.59, 31.19, 30.14, 23.98, 23.17; IR (cm^−1^): 3491, 3041, 1673, 1590, 1484, 1427, 1356, 1299, 1196, 1111, 882, 778, 743, 685; HR-MS: for C_19_H_19_ClN_2_O_3_ [M − H]^−^ calculated 357.1011 *m*/*z*, found 357.1017 *m*/*z*.

*Benzyl (2S)-2-[(4-chlorophenyl)carbamoyl]pyrrolidine-1-carboxylate* (**5c**) [58]. Yield 76%; m.p. 121–123 °C; HPLC pur. 98.79%; ${\left[\alpha \right]}_{D}^{25}$: −113.6 (*c* 1.0, CHCl_3_); ^1^H-NMR (DMSO-*d*_6_) δ: 10.17 (s, 1H), 7.56–7.68 (m, 2H), 7.27–7.44 (m, 5H), 7.08–7.26 (m, 2H), 5.04–5.14 (m, 1H), 4.90–4.98 (m, 1H), 4.29–4.40 (m, 1H), 3.42–3.57 (m, 2H), 2.12–2.34 (m, 1H), 1.79–1.99 (m, 3H); ^13^C-NMR (DMSO-*d*_6_) δ: 171.18, 170.89, 154.04, 153.67, 137.93, 137.84, 136.93, 136.73, 128.59, 128.55, 128.39, 128.07, 127.48, 126.91, 120.91, 120.74, 65.97, 65.90, 60.50, 60.00, 47.23, 46.59, 31.24, 30.17, 23.99, 23.18; IR (cm^−1^): 3270, 3184, 3064, 1704, 1673, 1542, 1445, 1359, 1300, 1167, 1117, 968, 840, 766, 732; HR-MS: for C_19_H_19_ClN_2_O_3_ [M − H]^−^ calculated 357.1011 *m*/*z*, found 357.1015 *m*/*z*.

*Benzyl (2S)-2-[(2-bromophenyl)carbamoyl]pyrrolidine-1-carboxylate* (**6a**) [59]. Yield 83%; m.p. 77–79 °C; HPLC pur. 98.82%; ${\left[\alpha \right]}_{D}^{25}$: −93.3 (*c* 1.0, CHCl_3_); ^1^H-NMR (DMSO-*d*_6_) δ: 9.43–9.60 (m, 1H), 7.60–7.68 (m, 2H), 7.23–7.43 (m, 6H), 7.14 (t, *J* = 7.3 Hz, 1H), 5.00–5.15 (m, 2H), 4.43–4.54 (m, 1H), 3.39–3.58 (m, 2H), 2.16–2.34 (m, 1H), 2.03 (td, *J* = 11.3, 4.4 Hz, 1H), 1.81–1.98 (m, 2H); ^13^C-NMR (DMSO-*d*_6_) δ: 171.07, 170.70, 154.30, 153.80, 136.90, 136.83, 135.86, 132.57, 128.36, 128.20, 127.21, 126.93, 126.49, 66.03, 65.96, 60.41, 59.76, 47.17, 46.58, 31.21, 29.91, 23.95, 23.09; IR (cm^−1^): 3239, 1717, 1670, 1522, 1414, 1353, 1201, 1125, 1029, 994, 728, 696; HR-MS: for C_19_H_19_BrN_2_O_3_ [M − H]^−^ calculated 401.0506 *m*/*z*, found 401.0513 *m*/*z*.

*Benzyl (2S)-2-[(3-bromophenyl)carbamoyl]pyrrolidine-1-carboxylate* (**6b**) [60]. Yield 75%; m.p. 124–126 °C; HPLC pur. 98.69%; ${\left[\alpha \right]}_{D}^{25}$: −92.0 (*c* 1.0, CHCl_3_); ^1^H-NMR (DMSO-*d*_6_) δ: 10.21 (s, 1H), 7.92–7.99 (m, 1H), 7.49 (dd, *J* = 16.7, 7.9 Hz, 1H), 7.38 (d, *J* = 4.7 Hz, 2H), 7.16–7.34 (m, 4H), 7.09–7.16 (m, 1H), 4.91–5.13 (m, 2H), 4.33 (ddd, *J* = 19.8, 8.4, 3.5 Hz, 1H), 3.40–3.55 (m, 2H), 2.17–2.30 (m, 1H), 1.80–1.98 (m, 3H); ^13^C-NMR (DMSO-*d*_6_) δ: 171.41, 171.12, 154.05, 153.64, 140.52, 140.40, 136.91, 136.72, 130.71, 130.64, 128.39, 128.02, 127.48, 126.96, 125.93, 125.87, 121.79, 121.50, 118.11, 117.94, 65.98, 65.91, 60.55, 60.04, 47.22, 46.58, 31.19, 30.14, 23.97, 23.17; IR (cm^−1^): 3254, 3173, 2878, 1701, 1668, 1591, 1418, 1352, 1258, 1128, 990, 874, 745, 667; HR-MS: for C_19_H_19_BrN_2_O_3_ [M − H]^−^ calculated 401.0506 *m*/*z*, found 401.0513 *m*/*z*.

*Benzyl (2S)-2-[(4-bromophenyl)carbamoyl]pyrrolidine-1-carboxylate* (**6c**) [60]. Yield 93%; m.p. 109–111 °C; HPLC pur. 99.10%; ${\left[\alpha \right]}_{D}^{25}$: −100.0 (*c* 1.0, CHCl_3_); ^1^H-NMR (DMSO-*d*_6_) δ: 10.17 (s, 1H), 7.43–7.63 (m, 4H), 7.27–7.42 (m, 2H), 7.08–7.26 (m, 3H), 4.90–5.12 (m, 2H), 4.29–4.39 (m, 1H), 3.35–3.58 (m, 2H), 2.13–2.31 (m, 1H), 1.76–1.99 (m, 3H); ^13^C-NMR (DMSO-*d*_6_) δ: 171.18, 170.90, 154.02, 153.64, 138.34, 138.24, 136.91, 136.71, 131.44, 128.37, 128.05, 127.46, 126.89, 121.28, 121.12, 65.95, 65.88, 60.51, 60.00, 47.20, 46.57, 31.21, 30.15, 23.97, 23.16; IR (cm^−1^): 3270, 1703, 1670, 1538, 1444, 1358, 1299, 1166, 1114, 1068, 1005, 967, 823, 731, 695; HR-MS: for C_19_H_19_BrN_2_O_3_ [M + H]^+^ calculated 403.0652 *m*/*z*, found 403.0658 *m*/*z*.

*Benzyl (2S)-2-{[2-(trifluoromethyl)phenyl]carbamoyl}pyrrolidine-1-carboxylate* (**7a**). Yield 65%; m.p. 82–84 °C; HPLC pur. 99.01%; ${\left[\alpha \right]}_{D}^{25}$: −100.0 (*c* 1.0, CHCl_3_); ^1^H-NMR (DMSO-*d*_6_) δ: 9.64 (d, *J* = 18.8 Hz, 1H), 7.56–7.76 (m, 2H), 7.26–7.52 (m, 6H), 7.14 (d, *J* = 7.6 Hz, 1H), 5.01–5.17 (m, 2H), 4.41–4.53 (m, 1H), 3.35–3.57 (m, 2H), 2.14–2.32 (m, 1H), 1.80–1.99 (m, 3H); ^13^C-NMR (DMSO-*d*_6_) δ: 171.86, 171.55, 153.81, 136.91, 135.25, 135.13 (q, ^4^*J_CF_* = 1.1 Hz, 2C), 132.99, 132.86, 129.97, 129.69 (q, ^4^*J_CF_* = 0.9 Hz, 2C), 128.37, 128.27, 127.77, 127.69, 127.46, 127.37, 126.82, 126.65, 126.26 (q, ^3^*J_CF_* = 5.2 Hz, 2C), 124.95 (q, ^2^*J_CF_* = 31.2 Hz, 1C), 124.94 (q, ^2^*J_CF_* = 30.3 Hz, 1C), 124.64 (q, ^3^*J_CF_* = 4.3 Hz, 2C), 123.56 (q, ^1^*J_CF_* = 273.7 Hz, 1C), 123.54 (q, ^1^*J_CF_* = 273.4 Hz, 1C), 122.65, 122.61, 66.01, 65.95, 60.17, 59.52, 47.15, 46.55, 30.99, 29.76, 23.85, 22.98; ^19^F NMR (DMSO-*d*_6_) δ: -59.24 (s, 1F), -59.31 (s, 1F); IR (cm^−1^): 3254, 1704, 1669, 1527, 1418, 1317, 1278, 1160, 1110, 1092, 909, 766, 691; HR-MS: for C_20_H_19_F_3_N_2_O_3_ [M − H]^−^ calculated 391.1275 *m*/*z*, found 391.1278 *m*/*z*.

*Benzyl (2S)-2-{[3-(trifluoromethyl)phenyl]carbamoyl}pyrrolidine-1-carboxylate* (**7b**). Yield 63%; m.p. 86–88 °C; HPLC pur. 99.54%; ${\left[\alpha \right]}_{D}^{25}$: −100.0 (*c* 1.0, CHCl_3_); ^1^H-NMR (DMSO-*d*_6_) δ: 10.38 (d, *J* = 4.8 Hz, 1H), 8.10 (d, *J* = 16.8 Hz, 1H), 7.72–7.85 (m, 1H), 7.49–7.62 (m, 1H), 7.27–7.46 (m, 3H), 6.99–7.24 (m, 3H), 4.85–5.18 (m, 2H), 4.36 (td, *J* = 8.7, 3.4 Hz, 1H), 3.36–3.59 (m, 2H), 2.14–2.37 (m, 1H), 1.76–2.05 (m, 3H); ^13^C-NMR (DMSO-*d*_6_) δ: 171.69, 171.41, 154.09, 153.67, 139.74, 139.64, 136.93, 136.73, 130.00, 129.95, 129.44 (q, ^2^*J_CF_* = 31.8 Hz, 1C), 129.41 (q, ^2^*J_CF_* = 31.8 Hz, 1C), 128.42, 127.97, 127.82, 127.53, 127.47, 126.99, 124.13 (q, ^1^*J_CF_* = 272.6 Hz, 2C), 122.86, 122.72, 119.67 (q, ^4^*J_CF_* = 1.0 Hz, 2C), 115.45 (q, ^3^*J_CF_* = 3.9 Hz, 2C), 115.25 (q, ^3^*J_CF_* = 3.9 Hz, 2C), 66.03, 65.95, 60.59, 60.10, 47.24, 46.61, 31.19, 30.15, 24.01, 23.20; ^19^F NMR (DMSO-*d*_6_) δ: −61.26 (s, 1F), −61.27 (s, 1F); IR (cm^−1^): 3270, 1694, 1674, 1545, 1419, 1325, 1165, 1123, 1111, 1063, 804, 740, 693; HR-MS: for C_20_H_19_F_3_N_2_O_3_ [M + H]^+^ calculated 393.1421 *m*/*z*, found 393.1424 *m*/*z*.

*Benzyl (2S)-2-{[4-(trifluoromethyl)phenyl]carbamoyl}pyrrolidine-1-carboxylate* (**7c**) [61]. Yield 62%; m.p. 106–109 °C; HPLC pur. 99.68%; ${\left[\alpha \right]}_{D}^{25}$: −98.8 (*c* 1.0, CHCl_3_); ^1^H-NMR (DMSO-*d*_6_) δ: 10.37–10.47 (m, 1H), 7.75–7.86 (m, 2H), 7.68 (d, *J* = 8.2 Hz, 2H), 7.35–7.40 (m, 1H), 7.28–7.35 (m, 1H), 7.14–7.23 (m, 2H), 7.06–7.13 (m, 1H), 5.07 (s, 1H), 4.90–4.97 (m, 1H), 4.32–4.43 (m, 1H), 3.41–3.57 (m, 2H), 2.16–2.34 (m, 1H), 1.79–1.99 (m, 3H); ^13^C-NMR (DMSO-*d*_6_) δ: 171.73, 171.44, 154.10, 153.68, 142.58, 142.52, 136.95, 136.73, 128.41, 128.06, 127.82, 127.51 (q, ^4^*J_CF_* = 1.0 Hz, 4C), 126.98, 126.00 (q, ^3^*J_CF_* = 3.9 Hz, 4C), 124.42 (q, ^1^*J_CF_* = 271.7 Hz, 2C), 123.41 (q, ^2^*J_CF_* = 31.8 Hz, 1C), 123.34 (q, ^2^*J_CF_* = 31.8 Hz, 1C), 119.24, 119.11, 66.03, 65.98, 60.58, 60.09, 47.24, 46.61, 31.22, 30.17, 24.02, 23.20; ^19^F NMR (DMSO-*d*_6_) δ: -60.19 (s, 1F); IR (cm^−1^): 3301, 1719, 1672, 1527, 1408, 1324, 1273, 1156, 1110, 1067, 835, 767, 696; HR-MS: for C_20_H_19_F_3_N_2_O_3_ [M + H]^+^ calculated 393.1421 *m*/*z*, found 393.1424 *m*/*z*.

*Benzyl (2S)-2-[(2-nitrophenyl)carbamoyl]pyrrolidine-1-carboxylate* (**8a**). Yield 72%; oil; HPLC pur. 99.71%; ${\left[\alpha \right]}_{D}^{25}$: −92.3 (*c* 1.0, CHCl_3_); ^1^H-NMR (DMSO-*d*_6_) δ: 10.46 (d, *J* = 14.1 Hz, 1H), 7.98 (d, *J* = 7.0 Hz, 1H), 7.56–7.85 (m, 2H), 7.11–7.45 (m, 6H), 4.93–5.18 (m, 2H), 4.31–4.49 (m, 1H), 3.37–3.63 (m, 2H), 2.12–2.38 (m, 1H), 1.79–2.07 (m, 3H); ^13^C-NMR (DMSO-*d*_6_) δ: 171.03, 170.77, 164.51, 154.32, 153.76, 141.83, 136.80, 136.68, 134.29, 134.10, 131.15, 128.37, 128.15, 127.15, 125.01, 124.68, 119.43, 66.13, 66.02, 60.69, 60.13, 47.16, 46.59, 30.59, 29.49, 23.90, 23.08; IR (cm^−1^): 3320, 2955, 2870, 1694, 1606, 1582, 1496, 1398, 1336, 1266, 1072, 866, 742, 691; HR-MS: for C_19_H_19_N_3_O_5_ [M + H]^+^ calculated 370.1398 *m*/*z*, found 370.1401 *m*/*z*.

*Benzyl (2S)-2-[(3-nitrophenyl)carbamoyl]pyrrolidine-1-carboxylate* (**8b**). Yield 60%; m.p. 101–104 °C; HPLC pur. 99.64%; ${\left[\alpha \right]}_{D}^{25}$: −104.3 (*c* 1.0, CHCl_3_); ^1^H-NMR (DMSO-*d*_6_) δ: 10.53 (d, *J* = 9.0 Hz, 1H), 8.52–8.77 (m, 1H), 7.93 (d, *J* = 6.6 Hz, 2H), 7.52–7.70 (m, 1H), 7.00–7.43 (m, 5H), 4.83–5.19 (m, 2H), 4.37 (dt, *J* = 8.2, 4.1 Hz, 1H), 3.39–3.63 (m, 2H), 2.12–2.40 (m, 1H), 1.74–2.04 (m, 3H); ^13^C-NMR (DMSO-*d*_6_) δ: 171.81, 171.53, 164.53, 153.63, 147.91, 147.87, 140.04, 139.93, 136.88, 136.65, 130.16, 130.11, 128.38, 127.99, 127.01, 125.30, 125.15, 117.87, 113.50, 113.34, 66.04, 65.97, 60.63, 60.15, 47.22, 46.55, 31.15, 30.11, 24.00, 23.20; IR (cm^−1^): 3270, 3087, 2983, 1706, 1681, 1598, 1520, 1446, 1394, 1296, 1243, 1123, 893, 739, 664; HR-MS: for C_19_H_19_N_3_O_5_ [M + H]^+^ calculated 370.1398 *m*/*z*, found 370.1400 *m*/*z*.

*Benzyl (2S)-2-[(4-nitrophenyl)carbamoyl]pyrrolidine-1-carboxylate* (**8c**) [62]. Yield 99%; m.p. 144–148 °C; HPLC pur. 98.52%; ${\left[\alpha \right]}_{D}^{25}$: −90.9 (*c* 1.0, CHCl_3_); ^1^H-NMR (DMSO-*d*_6_) δ: 10.67 (d, *J* = 6.8 Hz, 1H), 8.08–8.36 (m, 2H), 7.70–7.99 (m, 2H), 7.02–7.48 (m, 5H), 4.84–5.19 (m, 2H), 4.28–4.52 (m, 1H), 3.38–3.60 (m, 2H), 2.16–2.39 (m, 1H), 1.75–2.04 (m, 3H); ^13^C-NMR (DMSO-*d*_6_) δ: 172.02, 171.71, 164.52, 154.06, 153.58, 145.11, 145.02, 142.29, 136.87, 136.61, 128.38, 128.06, 127.48, 126.96, 124.95, 124.89, 119.02, 118.92, 66.03, 66.00, 60.63, 60.12, 47.20, 46.57, 31.13, 30.08, 23.99, 23.17; IR (cm^−1^): 3344, 2854, 1705, 1662, 1553, 1507, 1421, 1337, 1252, 1111, 986, 852, 696; HR-MS: for C_19_H_19_N_3_O_5_ [M + H]^+^ calculated 370.1398 *m*/*z*, found 370.1399 *m*/*z*.

General Procedure for Synthesis of Carbamates **9a**–**9c**: *N*-benzyloxycarbonyl-l-proline (0.50 g, 2.06 mmol) and dry TEA (0.81 g, 8.0 mmol) were dissolved in dry THF (7.5 mL) and cooled to 0 °C. To this solution, ethyl chloroformate (0.22 g, 2.06 mmol) was added dropwise during 15 min. The reaction mixture was then stirred for 30 min at the same temperature, and aminophenole (0.22 g, 2.06 mmol) was added. The resulting solution was stirred at 0 °C for 1 h and then at room temperature under inert atmosphere overnight. After reaction was finished, water (10 mL) was added, and the solution was three times extracted with diethyl ether. Combined organic layers were washed with 1M HCl, saturated NaHCO_3_ and brine and dried with anhydrous Na_2_SO_4_. Solvent was evaporated under reduced pressure, and the residue was triturated with diethyl ether/petroleum ether to give compounds **9a**–**9c**. All the studied compounds are presented in Table 1.

*Benzyl (2S)-2-[(2-hydroxyphenyl)carbamoyl]pyrrolidine-1-carboxylate* (**9a**) [61]. Yield 98%; oil; HPLC pur. 98.51%; ${\left[\alpha \right]}_{D}^{25}$: −101.3 (*c* 1.0, CHCl_3_); ^1^H-NMR (DMSO-*d*_6_) δ: 9.81 (br. s., 1H), 9.25 (s, 1H), 7.83 (dd, *J* = 13.8, 8.0 Hz, 1H), 7.12–7.43 (m, 6H), 6.70–6.99 (m, 2H), 4.95–5.21 (m, 2H), 4.45–4.65 (m, 1H), 3.38–3.57 (m, 2H), 2.06–2.39 (m, 1H), 1.77–2.04 (m, 3H); ^13^C-NMR (DMSO-*d*_6_) δ: 171.17, 170.74, 164.53, 153.87, 153.82, 147.53, 147.30, 136.84, 128.40, 128.11, 127.44, 126.84, 126.20, 124.45, 121.74, 118.92, 115.34, 115.25, 66.01, 65.86, 60.26, 59.89, 47.26, 46.66, 31.39, 30.02, 23.99, 23.14; IR (cm^−1^): 3658, 3282, 2944, 2870, 1767, 1663, 1597, 1526, 1452, 1414, 1348, 1165, 1103, 975, 746, 637; HR-MS: for C_19_H_20_N_2_O_4_ [M + H]^+^ calculated 341.1496 *m*/*z*, found 341.1500 *m*/*z*.

*Benzyl (2S)-2-[(3-hydroxyphenyl)carbamoyl]pyrrolidine-1-carboxylate* (**9b**) [63]. Yield 81%; m.p. 153–157 °C; HPLC pur. 98.61%; ${\left[\alpha \right]}_{D}^{25}$: −114.8 (*c* 1.0, CHCl_3_); ^1^H-NMR (DMSO-*d*_6_) δ: 9.90 (d, *J* = 17.9 Hz, 1H), 9.36 (d, *J* = 7.0 Hz, 1H), 7.29–7.42 (m, 3H), 6.93–7.25 (m, 5H), 6.41–6.51 (m, 1H), 4.93–5.13 (m, 2H), 4.21–4.41 (m, 1H), 3.38–3.56 (m, 2H), 2.24 (br. s., 1H), 1.77–1.98 (m, 3H); ^13^C-NMR (DMSO-*d*_6_) δ: 170.86, 170.55, 157.55, 154.00, 153.68, 139.96, 136.97, 136.79, 129.25, 128.38, 128.07, 127.46, 126.81, 110.39, 110.34, 110.08, 109.95, 106.52, 106.37, 65.90, 65.83, 60.46, 59.94, 47.22, 46.57, 31.30, 30.21, 23.95, 23.14; IR (cm^−1^): 3794, 3192, 2948, 2350, 1686, 1675, 1597, 1552, 1438, 1422, 1352, 1176, 1153, 1127, 1032, 1000, 973, 753, 687; HR-MS: for C_19_H_20_N_2_O_4_ [M + H]^+^ calculated 341.1496 *m*/*z*, found 341.1502 *m*/*z*.

*Benzyl (2S)-2-[(4-hydroxyphenyl)carbamoyl]pyrrolidine-1-carboxylate* (**9c**). Yield 60%; m.p. 193–194 °C; HPLC pur. 98.52%; ${\left[\alpha \right]}_{D}^{25}$: −70.7 (*c* 1.0, EtOH); ^1^H-NMR (DMSO-*d*_6_) δ: 9.76 (d, *J* = 14.1 Hz, 1H), 9.18 (d, *J* = 6.5 Hz, 1H), 7.28–7.43 (m, 4H), 7.12–7.27 (m, 3H), 6.65–6.73 (m, 2H), 4.93–5.13 (m, 2H), 4.25–4.36 (m, 1H), 3.39–3.55 (m, 2H), 2.12–2.28 (m, 1H), 1.77–1.97 (m, 3H); ^13^C-NMR (DMSO-*d*_6_) δ: 170.35, 170.10, 154.04, 153.77, 153.36, 137.01, 136.88, 130.69, 130.59, 128.40, 128.11, 127.48, 126.89, 121.21, 121.01, 115.01, 65.89, 60.45, 59.93, 47.25, 46.58, 31.35, 30.27, 23.99, 23.20; IR (cm^−1^): 3685, 3262, 2955, 1677, 1658, 1550, 1516, 1437, 1353, 1246, 1177, 1134, 995, 829, 691; HR-MS: for C_19_H_20_N_2_O_4_ [M + H]^+^ calculated 341.1496 *m*/*z*, found 341.1500 *m*/*z*.

### 3.3. In Vitro Evaluation of *AChE*- and *BChE*-Inhibiting Activity

The ability of all prepared compounds to inhibit AChE from electric eel (*Electrophorus electricus*) and BChE from equine serum (both purchased from Sigma, St. Louis, MO, USA) was determined in vitro using a modified Ellman’s method. The effectiveness of the inhibitors expressed as IC_50_ value represents the concentration of an inhibitor that is required for reduction of enzyme activity (or reaction rate) to 50% (sometimes it is referred to as the negative logarithm of the molar concentration inhibiting the enzyme activity by 50%, pIC_50_ = log 1/IC_50_). The Ellman’s method [64] is widely used for measuring cholinesterase activity and the effectivity of ChEIs. It is a simple, rapid and direct method to determine SH and –S–S– groups content in proteins [65]. Cholinesterase activity is measured indirectly by quantifying the concentration of 5-thio-2-nitrobenzoic acid (TNB) ion formed in the reaction between disulfide reagent 5,5′-dithiobis-2-nitrobenzoic acid (DTNB) and thiocholine, a product of substrate (i.e., acetylthiocholine, ATCh) hydrolysis catalyzed by cholinesterase [66].

All tested compounds were dissolved in DMSO (concentration 0.01 M) and diluted in demineralized water (concentrations 0.001 M and 0.0001 M). The ability of tested compounds to inhibit AChE (from electric eel) and BChE (from equine serum) was determined using the modified Ellman’s method at 25 °C in the presence of phosphate buffered saline (PBS, 0.1 M, pH 7.4) in a glass cuvette with 1 cm optical path. The enzyme activity in total reaction mixture (2 mL) was 0.2 U/mL, the concentration of substrate ATCh 40 μM and the concentration of DTNB 0.1 mM for all reactions. The IC_50_ value was obtained from the dependence of *v*_0_/*v*_i_ on the concentration of the tested compound (inhibitor), where v_0_ is the reaction rate of uninhibited reaction and v_i_ is the reaction rate of inhibited reaction (for the given concentration of the inhibitor). First, *v*_0_ was determined. Into the cuvette PBS (0.1 M, pH 7.4), DTNB and ATCh were placed. The enzymatic reaction was started by adding the enzyme. The dependence of absorbance (λ = 412 nm) on time was observed for 70 s (reference solution contained PBS, DTNB and ATCh), and then the reaction rate (*v*_0_) was calculated (*v* = Δ*A*/Δ*t*). The measurement was performed in triplicate at least, and average *v*_0_ was determined. Then, *v*_i_ (for the given concentration of the inhibitor) was determined. Into the cuvette DTNB, ATCh, a chosen volume of the suitably diluted inhibitor (to achieve the required concentration of the inhibitor in the total reaction mixture) and a certain volume of PBS (to achieve the total volume of the reaction mixture 2 mL after adding the enzyme) were placed. The enzymatic reaction was started by adding the enzyme. The dependence of absorbance (λ = 412 nm) on time was observed for 70 s (reference solution was the same as for uninhibited reaction), and then the reaction rate (*v*_i_) was calculated. For determination of IC_50_ values, twelve different concentrations of inhibitor were used and each measurement was performed in triplicate at least. Finally, the dependence of *v*_0_/*v*_i_ on the concentration of the inhibitor was determined, and IC_50_ was calculated from the obtained equation of the regression curve for y = 2 (coming out from the definition of IC_50_) [67]. The obtained results are summarized in Table 1.

### 3.4. Comparative Molecular Surface Analysis

A self-organizing neural network (SOM) is comprised of a single layer of neurons typically arranged as a hexagonal or rectangular array of nodes using the unsupervised learning rules initially proposed by Kohonen. The 2D topology of the neural grid with the defined winning and neighborhood distances between individual neurons directly specifies the mutual relations between the neurons. The presented multidimensional input vector $x_{s}=(x_{s1},\ldots ,x_{sm})$ is distributed between neurons according to the similarity/correlation weight criteria, where similar inputs are located in the same or proximal nodes. A classical competitive Kohonen (KNN) approach relies on the comparison of the input vector with the corresponding multi-element weight vectors $w_{j}=(w_{j1},\ldots ,w_{jm})$ that describe each neuron in order to select the winning one (out_C_)б into which a particular input will be projected. The winning neuron is detected by the optimization of the Euclidean distance between a weight (*w*) and a vector (*x*) according to the following formula:

(1) $$out_{C}=\min\left[{\sum_{i=1}^{m}\left(x_{i}-w_{ij}\right)}^{2}\right]$$

where j=1,…,n refers to a particular neuron, n refers to a number of neurons, m is the number of weights per neuron and s indicates a particular input. Contrary to “winner takes all”, in the “winner takes most” methodology, the weights of the winning neuron and its neighbors are then modified to resemble and subsequently attract similar input vectors. While the next input vector is being presented to the network, the entire procedure is repeated.

In fact, the self-organizing neural mapping is considered as a nonlinear projection tool, which reduces the dimensionality of the input object, e.g., converts 3D objects to 2D, while maintaining the topological relationships between input and output data. Additionally, the trained network can be employed for the projections of the specified molecular property prescribed to the input vector with the generation of the color-coded clustering planar pattern called a feature map. Consequently, the SOM algorithm was used to generate an electrostatic potential map as a 2D topographic pattern receiving input signals from points sampled randomly at the molecular surface [68]. In a such application, the specification of the closest neighbor and then projection of signals into this particular neuron is based on the comparison of each 3D input vector consisting of *x*, *y*, and *z* coordinates with a three-element weight vector describing each neuron. The shape of the certain molecular surface (template) encoded in the weights of the trained Kohonen network can be used for processing signals coming from the surface of other molecule(s) (counter-template) providing a series of comparative SOM maps to compare/contrast the superimposed molecular geometry.

The implementation of the SOM for the classification, visualization and compression of the structural data has been widely reported, in particular for 2D mapping of the electrostatic potential on 3D molecular surfaces or partial atomic charges for atomic molecular representation [69].

### 3.5. *PLS* Analysis

The partial least squares (PLS) method expresses the relation between the variable *y* and a set of predictors *X* in the form represented by the following Equation:

*y* = *X* × *b* + e (2)

where *b* is the vector of the regression coefficients and *e* is the vector of the errors. Generally, PLS models are constructed for centered/autoscaled data, and their complexity is estimated using, e.g., the leave-one-out cross-validation (LOO-CV) procedure. In the LOO-CV, one repeats the calibration process *m* times, each time treating the *i-*th left-out object as the prediction object. The dependent variable for each left-out object is calculated on the basis of the model with one, two, three, etc. factors. The root-mean-square error of CV for the model with *j* factors is defined as:

(3) $$RMSECV_{j}=\sqrt{\frac{\sum_{i}^{m}(obs_{i}-pred_{i,j}{)}^{2}}{m}}$$

where *obs* denotes the observed value of a dependent variable; *pred* is the predicted value of a dependent variable; and *i* refers to the object index, which ranges from 1 to *m*. A model with *k* factors, for which *RMSECV* reaches a minimum, is considered as the optimal one. The cross-validated $q_{CV}^{2}$ is calculated as:

(4) $$q_{CV}^{2}=1-\frac{\sum_{i}^{m}{\left(obs_{i}-pred_{i}\right)}^{2}}{\sum_{i}^{m}{\left(obs_{i}-mean(abs_{i})\right)}^{2}}$$

where *obs* is the observed value; *pred* is the predicted value; *mean* is the mean value of *obs*; and *i* refers to the object index, which ranges from 1 to *m*. The cross-validated standard error of prediction *s* amounts to:

(5) $$s=\sqrt{\frac{\sum_{i}{\left(obs_{i}-pred_{i}\right)}^{2}}{m-k-1}}$$

where *m* is the number of objects and *k* is the number of the PLS factors in the model.

The quality of external predictions was measured using the standard deviation of error of prediction (SDEP) and $q^{2}{}_{test}$ parameter, which are defined respectively as:

(6) $$SDEP=\sqrt{\frac{\sum_{i}^{n}{\left(pred_{i}-obs_{i}\right)}^{2}}{n}}$$

(7) $$q_{test}^{2}=1-\frac{\sum_{i}^{n}{\left(obs_{i}-pred_{i}\right)}^{2}}{\sum_{i}^{n}{\left(obs_{i}-mean(obs_{i})\right)}^{2}}$$

where *n* is the number of objects in a test set.

### 3.6. Iterative *PLS*-Based Variable Elimination

Redundant variables may influence a model and increase its complexity; therefore, the reduction in the number of variables facilitates the interpretation of the model considerably. To find only reliable variables that significantly contribute to the regression model, the modified PLS procedure with uninformative variable elimination (UVE-PLS) as well as its modification, namely, iterative variable elimination (IVE-PLS) can be applied successfully [70]. The original UVE-PLS algorithm, developed by Centner et al., analyzes the stability of regression coefficients expressed as the *mean*(*b*)/*s*(*b*) ratio, where *s*(*b*) represents the standard deviation of the regression coefficient *b* that is calculated by the PLS method [71]. Instead of a single step UVE-PLS procedure, we previously proposed an iterative algorithm based on the *abs*(*mean*(*b*)/*s*(*b*)) criterion to identify the variables to be eliminated. Basically, the entire procedure consists of the following steps: (i) standard PLS analysis with LOO–CV to assess the performance of the PLS model ($q_{CV}^{2}$); (ii) elimination of the matrix column with the lowest *abs(mean(b)*/*std(b))* value; (iii) standard PLS analysis of the new matrix without the column cancelled in Step (ii); and (iv) recurrent repetition of Steps i–iii to maximize the LOO $q_{CV}^{2}$ parameter.

### 3.7. *PCA* Analysis

The mapping of the molecular diversity forming the “infinite” chemical space (CS) into the corresponding biological or property space generally requires multi-dimensional descriptor representations. A specified molecule might be represented by a set of structural (S) and physicochemical (P) properties organized in a vector, which represents an object in the CS. The molecular distribution of the empirically (FCS) and virtually (VCS) generated compounds might be graphically investigated using, e.g., a linear projection procedure called Principal Component Analysis (PCA). PCA is a projection method that is designed to model multivariate data with a relatively small number of so-called principal components. PCs are constructed as a linear combination of original variables to maximize the description of data variance. The PCA model decomposes information contained in a data matrix into the principal component scores and loadings.

The score matrix contains information about any similarities among the data objects, while the loading matrix allows the similarities among the variables and their roles in the construction of a given principal component to be studied. The PCA model with *f* principal components for a data matrix *X* can be presented as follows:

*X* = *TP^T^* + *E* (8)

where *X* is a data matrix with *m* objects and *n* variables, *T* is the score matrix with dimensions (*m × f*), *P^T^* is a transposed matrix of loadings with dimensions (*f × n*) and E is a matrix of the residual variance (*m × n*) that is not explained by the first *f* principal components. The first few principal components often capture interesting information about the data structure and uncover groups of objects, atypical objects, etc., and also indicate the importance of the original data variables that contribute to the observed structure. Therefore, visualization of scores and loadings and their further simultaneous interpretation allow insight into a problem being studied to be gained [72].

### 3.8. Model Builder

The same laboratory was employed to specify all pharmacological data to eliminate potential data noise that might have been introduced by pooling of data sets coming from various sources. The in vitro AChE and BChE inhibition values (IC_50_) for the set of carbamate derivatives are listed in Table 1. The distributions of the IC_50_ inhibition of carbamates **1**–**9c** response in 12 equally spaced containers (expressed in μM) are presented as histograms in Figure 1. The CACTVS/csed molecular editor was used to specify the constitution of the respective compound models. The spatial geometry of molecules was produced using the 3D generator CORINA. The (inter)change file format converter OpenBabel was applied to convert the chemical data.

### 3.9. Molecular Modeling

The principal components of the modeling studies were conducted with the usage of the Sybyl–X 2.0/Certara software package (Certara, Princeton, NJ, USA) running on HP workstation with Debian 6.0 operating system. The standard Tripos force field (POWELL conjugate gradient algorithm) with 0.01 kcal/mol energy gradient convergence criterion and a distant dependent dielectric constant was used to optimize the initial geometry of each compound (MAXMIN2 module). The Gasteiger–Hückel procedure implemented in Sybyl for the electrostatic potential calculations was initially employed to calculate the partial atomic charges. The trial alignments are typically defined to systematically span the common scaffold of the analyzed compounds; therefore, one 15-ordered atom trial alignment on molecule **1** was selected to cover the entire bonding topology in the maximal common structure (MCS) by the atom FIT method based on the matching of atoms’ positions between the corresponding atom pairs.

The SONNIA software was employed in CoMSA analysis to simulate 20 × 20 to 50 × 50 SOMs with the winning distance varied in the range of 0.2–2.0. The Cartesian coordinates of the molecular surfaces for superimposed molecules were proceeded by the SOM network to form a 2D map of electrostatic potential. The structurally simplest analog **1** and molecules with the highest volume descriptor **7a**–**c** were used to form the template molecules. The output maps were subsequently transformed to a 400- to 2500-element vector, which was processed by PLS method implemented in the MATLAB programming environment.

The crystallographic structure of rhAChE was retrieved from the PDB repository (code 4EY6) containing two amino acid chains and two galanthamine molecules. All heteroatoms, including crystallographic waters, were extracted prior to the calculations. The ligand/protein structures were prepared for the docking study in the pdbqt file format with Gasteiger charges calculated. During the AutoDock simulation, various poses (default 9) were generated progressively from a single conformer (energy optimized molecule) by applying a collection of preferred torsion angles to the rotatable bonds and evaluated by united-atom scoring function. All predicted binding modes, including the positions of flexible side chains, were visualized using the VMD molecular graphics viewer.

### 3.10. In Vitro Cytotoxicity Assay

Human monocytic leukemia THP-1 cells were used for in vitro toxicity assay. Cells were obtained from the European Collection of Cell Cultures (ECACC, Salisbury, UK) and routinely cultured in RPMI 1640 (Lonza, Verviers, Belgium) medium supplemented with 10% fetal bovine serum (FBS, Sigma), 2% l-glutamine, 1% penicillin and streptomycin (Lonza, Verviers, Belgium) at 37 °C with 5% CO_2_. Cells were passaged at approximately one week intervals. The cytotoxicity of the compounds was determined using a WST-1 assay kit (Roche Diagnostics, Mannheim, Germany) according to the manufacturer’s instructions. The tested compounds were dissolved in DMSO and added in five increasing concentrations (0.37, 1.1, 3.3, 10, and 30 μM) to the cell suspension in the culture RPMI 1640 medium. The maximum concentration of DMSO (Sigma) in the assays never exceeded 0.1%. Subsequently, the cells were incubated for 24 h at 37 °C with 5% CO_2_. For WST-1 assays, cells were seeded into 96-well plates (5 × 10^4^ cells/well in 100 μL culture medium) in triplicate in serum-free RPMI 1640 medium, and measurements were taken 24 h after the treatment with the compounds. The median inhibition concentration values, IC_50_, were deduced through the production of a dose-response curve. All data were evaluated using the GraphPad Prism 5.00 software (GraphPad Software, San Diego, CA, USA). The results are summarized in Table 1.

## 4. Conclusions

Twenty-five benzyl (2*S*)-2-(arylcarbamoyl)pyrrolidine-1-carboxylates were synthesized and characterized by IR, ^1^H, ^13^C and ^19^F NMR spectroscopy and HRMS as well as by optical rotations. All compounds were tested for their in vitro ability to inhibit AChE and BChE. The selectivity index of individual compounds to cholinesterases was determined. The screening of the cytotoxicity of all the compounds was performed using human THP-1 cells, no significant changes in the viability of cells were found up to concentration 30 μM. All the compounds showed rather moderate inhibitory effect against AChE; benzyl (2*S*)-2-[(2-chlorophenyl)carbamoyl]pyrrolidine-1-carboxylate (**5a**) (IC_50_ = 46.35 μM) was the most potent agent. On the other hand, benzyl (2*S*)-2-[(2-bromophenyl)-(**6a**) and benzyl (2*S*)-2-[(4-bromophenyl)-carbamoyl]pyrrolidine-1-carboxylate (**6c**) expressed anti-BChE activity (IC_50_ = 27.38 and 28.21 μM, respectively) comparable with that of rivastigmine. Compound **6a** and benzyl (2*S*)-2-[(2-hydroxyphenyl)carbamoyl]pyrrolidine-1-carboxylate (**9a**) demonstrated greater selectivity to BChE. A comparative receptor-independent structure–inhibitory activity study of proline-based carbamates as potential ChEIs is reported using the 3D neural methodology (CoMSA) coupled with the IVE-PLS procedure. In fact, the ability of the fuzzy molecular representation for a variety of training/test subset distribution was examined for a large populations models generated using the stochastic SMV procedure. A systematic space inspection merged with the variable elimination method produce the probabilistic pharmacophore geometry specifying descriptors that have potentially the highest individual weightings to the observed AChE/BChE profiles. In silico activity examination confirmed the significant qualitative difference in the inhibitory potency of positional isomers reflected in the empirical data, especially for unfavorable *para*-substitution of the phenyl ring. The visual investigation of a pharmacophore pattern gives a simplified picture of regions that can be modified to modulate the desired BChE activity of the compound, providing valuable hints for the property-oriented synthesis. Moreover, PCA analysis was employed to illustrate the crucial variations in the inhibitory efficiency of the screened molecules with respect to their structure, lipophilicity and activity profile. The performed molecular docking study demonstrated the importance of the side chain R, especially for *ortho*-positioned analogs directly attached to the phenyl ring. Moreover, a chlorine atom can contribute to hydrogen bond interactions with Tyr337 of chain A as postulated in the case of the galanthamine binding mode. The inhibition activity profile for the positional isomers can be partially explained by the possibility of hydrogen bond interactions in the catalytic core ranked according to *ortho* > *meta* > *para* substitution. It seems that an increase of bulkiness at the *para* position of the phenyl ring is unfavorable for the inhibitory potency of the investigated carbamates as observed also in the pharmacophore study.

## Figures and Tables

**Table 1:** Structures of discussed carbamates **1**–**9c**; calculated values of lipophilicity (clogP), polar surface area (PSA), polar volume (PV) and in vitro AChE and BChE inhibition (IC_50_) in comparison with standards rivastigmine (RIV) and galanthamine (GLT) and in vitro cytotoxicity assay (IC_50_) of compounds. ChE inhibitions are expressed as mean ± SD (*n* = 3 experiments).

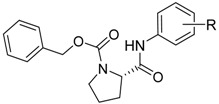								
Comp.	R	clogP	PSA (Å)	PV	AChE IC_50_ (μM)	BChE IC_50_ (μM)	SI *	tox. IC_50_ (μM)
**1**	H	4.1632	49.446	148.212	105.88 ± 2.20	55.57 ± 1.01	1.91	>30
**2a**	2-OCH_3_	3.6486	51.491	160.579	130.99 ± 1.29	51.53 ± 0.75	**2.54**	>30
**2b**	3-OCH_3_	4.2386	61.506	165.704	126.13 ± 2.79	58.47 ± 0.42	2.16	>30
**2c**	4-OCH_3_	4.2386	61.876	148.051	109.36 ± 0.22	117.40 ± 0.45	0.93	>30
**3a**	2-CH_3_	4.0122	45.255	141.705	61.94 ± 1.35	98.27 ± 5.88	0.63	>30
**3b**	3-CH_3_	4.6622	49.460	129.946	81.06 ± 0.03	114.84 ± 4.83	0.71	>30
**3c**	4-CH_3_	4.6622	49.466	132.090	123.41 ± 0.44	153.11 ± 2.29	0.81	>30
**4a**	2-F	3.9638	48.435	144.032	51.87 ± 0.76	59.97 ± 0.00	0.86	>30
**4b**	3-F	4.5638	49.474	131.146	69.88 ± 0.21	34.61 ± 0.11	2.02	>30
**4c**	4-F	4.5638	49.441	144.623	87.23 ± 1.00	58.14 ± 1.39	1.50	>30
**5a**	2-Cl	4.2838	48.725	135.310	46.35 ± 1.05	53.47 ± 0.52	0.87	>30
**5b**	3-Cl	5.1338	49.486	134.686	48.27 ± 0.11	64.20 ± 7.14	0.75	>30
**5c**	4-Cl	5.1338	49.435	133.841	77.34 ± 3.80	147.34 ± 1.18	0.52	>30
**6a**	2-Br	4.4038	49.183	137.829	84.14 ± 4.39	27.38 ± 0.85	**3.07**	>30
**6b**	3-Br	5.2838	49.440	137.166	74.16 ± 0.20	47.68 ± 0.08	1.56	>30
**6c**	4-Br	5.2838	49.462	133.509	81.32 ± 0.67	28.21 ± 0.50	**2.88**	>30
**7a**	2-CF_3_	4.0470	42.920	142.558	73.24 ± 2.88	44.89 ± 1.66	1.63	>30
**7b**	3-CF_3_	5.4970	49.450	118.742	68.91 ± 0.06	39.46 ± 1.40	1.75	>30
**7c**	4-CF_3_	5.4970	49.438	124.821	88.65 ± 1.60	75.48 ± 2.94	1.17	>30
**8a**	2-NO_2_	0.8712	110.799	195.483	152.21 ± 0.01	48.69 ± 0.80	3.13	>30
**8b**	3-NO_2_	0.8712	134.854	207.158	64.99 ± 0.97	55.93 ± 0.63	1.16	>30
**8c**	4-NO_2_	0.8712	134.702	209.563	84.52 ± 0.06	58.75 ± 0.27	1.44	>30
**9a**	2-OH	3.7262	93.856	164.611	153.40 ± 9.97	42.01 ± 1.26	**3.65**	>30
**9b**	3-OH	3.4962	105.477	172.921	122.00 ± 1.38	86.22 ± 0.40	1.41	>30
**9c**	4-OH	3.4962	105.171	173.231	91.08 ± 0.27	132.97 ± 0.39	0.68	>30
**RIV**	–	–	–	–	50.10 ± 3.08	19.95 ± 0.31	25.11	–
**GLT**	–	–	–	–	4.0 ± 0.13	7.96 ± 0.59	0.50	–

* SI (index selectivity) = IC_50_ (AChE)/IC_50_ (BChE). Greater selectivity to BChE in bold.

## Supplementary Materials

The supplementary material is available online.
