# Supplementary material for: Proline-Based Carbamates as Cholinesterase Inhibitors
Source: Molecules. 2017 Nov 14;22(11):1969. doi: 10.3390/molecules22111969 (PMC6150311; doi:10.3390/molecules22111969)
Supplement: Supplementary file 1 [file molecules-22-01969-s001.pdf]

Supplementary Materials

Table S1.

L.p.	Polar surface area	Polar volume	Surface area	Volume	Acceptor count (sybyl)	Atom count	Bond count	Donor count (sybyl)	Hydrophobe count	Rotatable Bond count	Acceptor Mount (Lipinski)	ClogP	Donor Mount (Lipinski)	Lipinski violations	Molecular weight
**1**	49.446	148.212	605.397	1006.661	3	44	46	1	3	6	5	4.1632	1	0	324.3737
**2a**	51.491	160.579	648.635	1063.791	4	48	50	1	3	7	6	3.6486	1	0	354.3997
**2b**	61.506	165.704	653.223	1083.814	4	48	50	1	3	7	6	4.2386	1	0	354.3997
**2c**	61.876	148.051	653.419	1073.462	4	48	50	1	3	7	6	4.2386	1	0	354.3997
**3a**	45.255	141.705	623.038	1038.938	3	47	49	1	3	6	5	4.0122	1	0	338.4003
**3b**	49.460	129.946	636.949	1050.936	3	47	49	1	3	6	5	4.6622	1	0	338.4003
**3c**	49.466	132.090	637.767	1050.962	3	47	49	1	3	6	5	4.6622	1	0	338.4003
**4a**	48.435	144.032	610.640	1018.047	3	44	46	1	3	6	5	3.9638	1	0	342.3642
**4b**	49.474	131.146	614.901	1012.274	3	44	46	1	3	6	5	4.5638	1	0	342.3642
**4c**	49.441	144.623	614.922	1021.213	3	44	46	1	3	6	5	4.5638	1	0	342.3642
**5a**	48.725	135.310	618.371	1030.333	3	44	46	1	3	6	5	4.2838	1	0	358.8188
**5b**	49.486	134.686	628.707	1042.689	3	44	46	1	3	6	5	5.1338	1	1	358.8188
**5c**	49.435	133.841	628.664	1038.846	3	44	46	1	3	6	5	5.1338	1	1	358.8188
**6a**	49.183	137.829	621.311	1038.864	3	44	46	1	3	6	5	4.4038	1	0	403.2698
**6b**	49.440	137.166	633.526	1053.128	3	44	46	1	3	6	5	5.2838	1	1	403.2698
**6c**	49.462	133.509	633.536	1046.658	3	44	46	1	3	6	5	5.2838	1	1	403.2698
**7a**	42.920	142.558	636.372	1076.084	3	47	49	1	4	6	5	4.0470	1	0	392.3717
**7b**	49.450	118.742	659.789	1088.469	3	47	49	1	4	6	5	5.4970	1	1	392.3717
**7c**	49.438	124.821	660.914	1079.248	3	47	49	1	4	6	5	5.4970	1	1	392.3717
**8a**	110.799	195.483	623.940	1045.108	5	46	48	1	3	7	8	0.8712	1	0	369.3713
**8b**	134.854	207.158	641.084	1063.287	5	46	48	1	3	7	8	0.8712	1	0	369.3713
**8c**	134.702	209.563	641.602	1057.242	5	46	48	1	3	7	8	0.8712	1	0	369.3713
**9a**	93.856	164.611	614.104	1021.122	4	45	47	2	3	6	6	3.7262	2	0	340.3731
**9b**	105.477	172.921	619.746	1034.721	4	45	47	2	3	6	6	3.4962	2	0	340.3731
**9c**	105.171	173.231	619.497	1023.670	4	45	47	2	3	6	6	3.4962	2	0	340.3731
